# Measuring Subjective Sleep Quality: A Review

**DOI:** 10.3390/ijerph18031082

**Published:** 2021-01-26

**Authors:** Marco Fabbri, Alessia Beracci, Monica Martoni, Debora Meneo, Lorenzo Tonetti, Vincenzo Natale

**Affiliations:** 1 Department of Psychology, University of Campania Luigi Vanvitelli, 8100 Caserta, Italy; alessia.beracci@unicampania.it; 2 Department of Experimental, Diagnostic and Specialty Medicine, University of Bologna, 40138 Bologna, Italy; monica.martoni@unibo.it (M.M.); debora.meneo@studio.unibo.it (D.M.); 3 Department of Psychology “Renzo Canestrari”, University of Bologna, 40127 Bologna, Italy; lorenzo.tonetti2@unibo.it (L.T.); vincenzo.natale@unibo.it (V.N.)

**Keywords:** sleep quality, psychometric properties, self-report questionnaire, dimensionality

## Abstract

Sleep quality is an important clinical construct since it is increasingly common for people to complain about poor sleep quality and its impact on daytime functioning. Moreover, poor sleep quality can be an important symptom of many sleep and medical disorders. However, objective measures of sleep quality, such as polysomnography, are not readily available to most clinicians in their daily routine, and are expensive, time-consuming, and impractical for epidemiological and research studies., Several self-report questionnaires have, however, been developed. The present review aims to address their psychometric properties, construct validity, and factorial structure while presenting, comparing, and discussing the measurement properties of these sleep quality questionnaires. A systematic literature search, from 2008 to 2020, was performed using the electronic databases PubMed and Scopus, with predefined search terms. In total, 49 articles were analyzed from the 5734 articles found. The psychometric properties and factor structure of the following are reported: Pittsburgh Sleep Quality Index (PSQI), Athens Insomnia Scale (AIS), Insomnia Severity Index (ISI), Mini-Sleep Questionnaire (MSQ), Jenkins Sleep Scale (JSS), Leeds Sleep Evaluation Questionnaire (LSEQ), SLEEP-50 Questionnaire, and Epworth Sleepiness Scale (ESS). As the most frequently used subjective measurement of sleep quality, the PSQI reported good internal reliability and validity; however, different factorial structures were found in a variety of samples, casting doubt on the usefulness of total score in detecting poor and good sleepers. The sleep disorder scales (AIS, ISI, MSQ, JSS, LSEQ and SLEEP-50) reported good psychometric properties; nevertheless, AIS and ISI reported a variety of factorial models whereas LSEQ and SLEEP-50 appeared to be less useful for epidemiological and research settings due to the length of the questionnaires and their scoring. The MSQ and JSS seemed to be inexpensive and easy to administer, complete, and score, but further validation studies are needed. Finally, the ESS had good internal consistency and construct validity, while the main challenges were in its factorial structure, known-group difference and estimation of reliable cut-offs. Overall, the self-report questionnaires assessing sleep quality from different perspectives have good psychometric properties, with high internal consistency and test-retest reliability, as well as convergent/divergent validity with sleep, psychological, and socio-demographic variables. However, a clear definition of the factor model underlying the tools is recommended and reliable cut-off values should be indicated in order for clinicians to discriminate poor and good sleepers.

## 1. Introduction

The term sleep quality is commonly used in sleep medicine and can refer to a collection of sleep measures including Total Sleep Time (TST), Sleep Onset Latency (SOL), sleep maintenance, Total Wake Time (TWT), Sleep Efficiency (SE), and sometimes sleep disruptive events such as spontaneous arousal or apnea [1]. Moreover, sleep quality appears to be orthogonal to the term sleep quantity. For example, the presence of sleep complaints has been reported even when SOL, Wakefulness After Sleep Onset (WASO), TST and awakening were similar to those reported in normal non-complaining individuals [2]. Complaints of disturbed (or poor quality) sleep have been confirmed in almost every country [3] and among patients in all specialties of medicine [4,5,6,7,8,9,10,11,12]. Untreated sleep disorders may lead to potentially life-threatening symptoms, considering that sleep disorders are not only a consequence of medical illness but are also primary drivers of other illnesses [13]. It is now recognized that sleep disturbances are associated with neurocognitive dysfunctions, attention deficits, impaired cognitive performance, depression, anxiety, stress, and poor impulse controls [11]. Poor sleep can severely affect daytime performance, both socially and at work, and increases the risk of occupational and automobile accidents, poor quality of life and poor overall health [14]. Thus, the assessment of sleep quality appears to be relevant for epidemiological and clinical studies.

Sleep quality can be assessed using both objective and subjective methods. Objective methods such as polysomnography (PSG) and actigraphy demonstrate high reliability in obtaining information on sleep parameters [1]. However, these objective methods, such as PSG (see also Multiple Sleep Latency Test or MSLT for the assessment of daytime sleepiness), are not readily available to most clinicians in their daily routine, and are expensive and time-consuming [15]. Even if the actigraph has several advantages (e.g., it is not costly), the recorded activity is only a proxy for sleep and is not sleep itself. Moreover, there are a variety of devices and scoring algorithms available that limit the comparability between different actigraphic devices [1].

Among the subjective methods, the sleep diary is the most widely-used assessment [16]. The sleep diary requires the client to record daily morning estimates for the parameters of their sleep pattern, and, as such, yields information concerning a number of relevant metrics such as SOL, WASO, TST, total time spent in bed (TIB), SE, and satisfaction as a subjective global appraisal of each night’s sleep [16]. However, it is clear that its successful use relies heavily on daily (prospective) recordings as soon as individuals wake up in the morning, a task that may be difficult for older people to remember to do consistently, limiting the utility of the sleep diary for screening or epidemiological studies. In contrast, retrospective self-report measures, such as questionnaires, can be widely used in both routine care and clinical trials considering that they have several advantages including their low cost, and their potential to be administered to several types of populations over the Internet [17], as these measures are self-explanatory and do not require supervision. In addition, self-rating questionnaires have the advantages of high patient compliance, ease of administration, and reduced demand on medical specialists’ time.

Given the important diagnostic role of rating scale questionnaires, it is beyond doubt that the psychometric properties of these tools need to be established. Specifically, in the present review, we consider dimensionality, reliability, and construct validity [18,19,20]. Dimensionality is generally evaluated by factor analysis (e.g., exploratory factor analysis, or EFA, and confirmatory factor analysis, or CFA), which attempts to discover patterns in a set of variables based on shared variance. In particular, this analysis tries to identify the simplest and most parsimonious means with which to interpret and represent the observed data, in order to infer the smallest number of unobserved or latent variables that can still account for the observable variables. Indeed, EFA is used to find the smallest number of common latent factors, accounting for the correlations [20], while CFA is further used to test the relationship between the observed data and the hypothesized latent factors [20]. Reliability reflects the extent to which the measure is reliable, that is, free from errors in the scores that are not due to the true state of the construct measured. It can be expressed as internal consistency (Cronbach’s alpha or α), test-retest (e.g., intraclass correlation coefficient or ICC), inter-rater (degree of agreement between the scores given by different raters for the same respondent) or intra-rater (degree of agreement between scores given by the same respondent or rater at one time and those given at another time) reliability. Cronbach’s α ranges from 0 to 1 and a higher score indicates greater internal consistency [18]. Test-retest reliability calculates the absolute changes in a measure assessed independently on two distinct occasions [19]. The ICC is the preferred method to assess test-retest reliability and it is a measure of the agreement between two (or more) raters or evaluation methods in the same set of participants. The ICC ranges from 0 to 1 and higher values represent greater agreement between raters or evaluations [19]. Construct validity indicates the degree to which the measure scores reflect the hypotheses, including convergent (the degree of relatedness between two or more constructs hypothesized to be related), divergent (the degree of relatedness between two or more constructs hypothesized to be different) and known-group (the ability of the measure to discriminate between a group of individuals known to have a particular trait and those who do not have that trait). Finally, when available, the sensibility to change is an important piece of information related to how much the sleep questionnaire is able to detect the improvement or the effect of a specific therapy on sleep disorders.

The importance of reviewing the reliability, validity, and dimensionality of questionnaires assessing sleep quality for research, epidemiological, and clinical studies is shown by the strong relationship between reliability (i.e., whether the items of a scale measure the same construct) and validity (i.e., whether or not a scale measures what it is intended to measure). Although reliability is important for a study, internal consistency is not sufficient if it is not combined with validity. Thus, for a test to be reliable, it also needs to be valid. The capacity of a sleep questionnaire to screen for poor and good sleepers is related to its construct validity. The Receiver Operating Characteristic (ROC) curve analysis is usually used to determine a cut-off value. In the ROC curve analysis, the sensitivity (i.e., the probability of a positive screen result, that is, the proportion of accurately classified individuals who report poor sleep quality) and specificity (i.e., the probability of a negative screen result, that is, the proportion of accurately classified individuals who have good sleep quality) are plotted against each other. The Area Under a ROC Curve (AUC) provides a measure of the discriminatory power of a screening test at a single threshold that separates poor and good sleepers. The AUC ranges from 0 to 1 and, thus, a value of 0.5 indicates a lack of effectiveness while a value that is very close to 1 indicates a very efficient tool.

Moreover, the dimensionality of the questionnaires reflects whether the items are all correlated and representative of factors. Indeed, the consequence of a questionnaire being heterogeneous or multidimensional may be a possible attenuation of its practical application in clinical diagnostics. The dimensionality of a questionnaire directly influences the reporting of its intended measures. For example, if a questionnaire is supposed to be described with one single factor, suggesting the practical usefulness of its total score for screening individuals, but the factor analysis shows that a 2- or 3-factor model obtains a better fit with data, then the diagnostic use of the total score is in question. In the present review, we decided to report information, for each article included, regarding these three psychometric features: dimensionality, reliability, and validity.

As reported by Buysse and colleagues [21], sleep quality represents a complex construct that is difficult to define. In line with the clinical sleep dysfunction evaluation [11], the main complaints of a patient are the inability to obtain an adequate nighttime sleep even when there is the opportunity for sleep (i.e., insomnia disorder), negative daytime consequences due to poor sleep (e.g., daytime sleepiness), episodic nocturnal movements or behaviors (e.g., snoring, kicking of legs, bruxism, sleep walking, or talking), or a combination of these concerns. Thus, the self-reported questionnaires assessing poor sleep may incorporate all items (or a combination of them) of the aforementioned aspects, or may be selective for the assessment of specific sleep problems (e.g., insomnia or daytime sleepiness).

In line with these assumptions, to the best of our knowledge, no reviews have been published that concurrently consider dimensionality, reliability, and validity of the tools assessing subjective sleep quality. At the end of 2007, Martoni and Biagi [22] published a review reporting 26 possible sleep quality questionnaires (see Table 2, p. 323) [22], while the majority of the published reviews focuses on a few tools [e.g., 4–5,7,11–12], limiting any comparisons. For example, Wells et al. [4] considered four questionnaires, Hoey et al. [6] took into account three subjective sleep measurement scales, while Mollayeva et al. [11] focused on one tool only. However, the review by Martoni and Biagi [22], while more comprehensive, was published in Italian and an update of this review is needed. For this reason, we decided to review the psychometric properties and the dimensionality of all sleep questionnaires reported by Martoni and Biagi [22] in studies published within the temporal range of 2008 to 2020, in adults, and in clinical and non-clinical populations.

## 2. Materials and Methods

### Identification of Eligible Studies

PubMed and SCOPUS were searched from 1 January 2008 to 30 June 2020 for each questionnaire presented in [22]. Filters were applied to the search, limiting the selection to those studies involving humans with age > 18 years and published in English. Of the papers located, reference lists were also scanned for further papers and a search was undertaken to discover any papers related to the aim of the present review.

**Figure 1:**
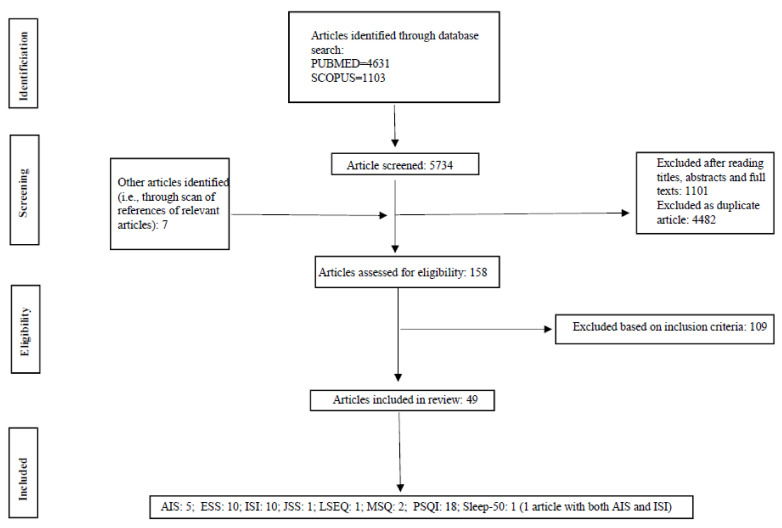
Flow chart documenting process of article selection for review.

The following descriptors and medical subject reading (MeSH) terms were used as search terms in the databases: “the extended name of the questionnaire” (e.g., Mini-Sleep Questionnaire) OR “acronym form” (e.g., MSQ) AND “reliability” OR “reliable” OR “test-retest” OR “validity” OR “validation” OR “valid”. This procedure was adopted for all questionnaires reported in Table 2 (p. 323) of [22]. A total of 5734 articles were initially identified (Figure 1). Through the process of article screening, 49 articles, referring to eight questionnaires for the assessment of sleep quality, were included in the final analysis. These articles respected our inclusion criteria: (1) the study population was composed of adults with age > 18 years; (2) the articles were published in English in the temporal range of 2008 to 2020; (3) the study was original research reporting reliability (Cronbach’s alpha and/or test-retest and/or split-half reliability), validity (convergent/divergent correlations and/or known-group differences), and dimensionality of a specific sleep tool (Figure 1). According to the aforementioned three points, the questionnaires included in the present review were: Pittsburgh Sleep Quality Index (PSQI), Athens Insomnia Scale (AIS), Insomnia Severity Index (ISI), Mini-Sleep Questionnaire (MSQ), Jenkins Sleep Scale (JSS), Leeds Sleep Evaluation Questionnaire (LSEQ), SLEEP-50 Questionnaire (SLEEP-50), and Epworth Sleepiness Scale (ESS).

Data extraction:

Potentially relevant papers were selected by (1) screening the titles, (2) screening the abstracts, and (3) retrieving and screening the full article to determine whether it met the inclusion criteria when the abstract did not provide sufficient data or was not available. The literature screenings were performed by two authors (F. M. and M. D.) independently using a pre-defined study extraction form and the results were compared. When a disagreement occurred, the article was evaluated by a third author (M. M.) blinded to the issue of the disagreement. The publication data included study characteristics: authors, tool name and its acronym, publication year, population type, sample size, number of females, and mean age in years of the general sample or of specific groups involved in the study. The definition of the construct, the structure (items, response format, etc.) of the questionnaire, the temporal period assessed, and the any translated versions were also recorded. Finally, the reliability coefficients, test-retest values, construct validity (convergent and divergent correlations with other measures, as well as known-group differences), the ROC curve analysis and eventually cut-off values were extracted. Finally, summaries of the exploratory, confirmatory, or principal component analysis (PCA, that is, the extraction of linear composites of observed variables) were indicated. A descriptive analysis of the articles was performed for the measures extracted.

## 3. Results

### 3.1. The Most Commonly Used Tool: PSQI

The Pittsburgh Sleep Quality Inventory (PSQI; [21]) is the most commonly used measure of subjective self-report sleep quality for two main reasons. It was not only developed to quantify sleep quality [21] but also, in the majority of studies that validate a sleep questionnaire, the PSQI has been used as convergent validity, suggesting that the PSQI can be considered as an accepted reference or gold standard for self-perceived sleep quality. In addition, it is the most widely used sleep health assessment tool in both clinical and non-clinical populations [11]. In the present review, it was the questionnaire with the highest number of studies investigating its psychometric properties, beyond factor structure. The PSQI consisted of 24 questions or items to be rated, relating to the past month (0–3 for 20 items while 4 items were open-ended), 19 of which were self-reported and 5 of which required secondary feedback from a room or bed partner. Only 19 items (15 rated 0–3 and 4 open-ended) were used for the evaluation of sleep quality as perceived by the individuals. The open-ended items were also scored as categorical values (rated 0–3) as per the range of values reported by the patients. These 19 self-reported items were then used to generate scores, which ranged from 0 (no difficulty) to 3 (severe difficulty), representing the PSQI’s seven components: sleep quality (C1), sleep latency (C2), sleep duration (C3), habitual sleep efficiency (C4), sleep disturbance (C5), use of sleep medications (C6), and daytime disturbance (C7). The scores for each component were summed to get a total score, also termed the global score (range 0–21), providing an efficient summary of the respondent’s sleep experience and quality. Panayides et al. [23] not only revised the original 4-point Likert scale with a more optimal 3-point Likert scale that is more appropriate for a non-clinical sample, but also proposed a 16-item version following two calibrations using the Rasch model [24]. In contrast, Chien et al. [25] proposed a revised PSQI: short form Chinese version or SC_PSQI with nine items (sleep latency, habitual sleep efficiency, sleep disturbances, sleep interruptions, use of sleeping medication, daytime dysfunction, days of insomnia, fatigue upon awakening in bed, and earlier awakening). In addition, scoring of answers was changed from 0–3 to 0–2, and the score total amounted to 18.

Among different sample types (from non-clinical individuals to different medical populations), with a vast range of numerosity (from 50 to 3.742) comprising a wide age range (18–80+) and different language versions (English, Chinese, Greek, Korean, Italian, Spanish, Sinhala, European Portuguese, Malay, Kurdish, and Arabic), the most interesting result is related to the factor structure underlying the PSQI, using different factorial analyses (Table 1). In the present review, six papers [23,25,26,27,28,29] reported unidimensionality, six studies indicated a 2-factor model [30,31,32,33,34,35] and two investigations found a three-factor model [36,37]. The remaining articles in Table 1 did not show a unique factor model [38,39,40,41]. In the two-factor solution, the C2, C3, and C4 loaded on one factor (i.e., a sort of sleep efficiency factor) whereas C5 and C7 loaded on the other factor (i.e., a version of the daytime disturbance factor). The C1 was often an added component of the factor containing C2–C4 while C6 either added to a factor comprising C5 and C7 or was deleted because of low (<0.40) loading value [30,31,32,33,34]. The only exception was the study conducted in cancer patients [35] with a factor labelled Perceived Sleep Quality with C1, C2, C5, C6, and C7, and the other factor labelled Sleep Efficiency with C3 and C4. Inter-factor correlation was on average 0.476. By contrast, the three-factor solution indicated that the Sleep Efficiency factor included C3 and C4, the Perceived Sleep Quality factor included C1, C2 and C6, and the Daily Disturbance factor included C5 and C7, with correlations between first and second factors (mean 0.465), between second and third factors (mean 0.58), and between first and third factors (mean 0.415) [26,33,36,39]. Alternative models included the same factors with the exclusion of C6. Importantly, a single study reported a different three-factor structure for male and female adults [41]. Specifically, for men F1 was determined by C1, C3, and C4, F2 by C5 and C7, and F3 included C2 and C6; for women F1 was determined by C1, C5, and C7, F2 included C3 and C4, and F3 was loaded by C2 and C6. This result could indicate the presence of gender differences in sleep quality.

As shown in Table 1, the Cronbach’s alpha was on average equal to 0.76 [42]. It should be noted that the Cronbach’s alpha increased, on average, by 2 points, excluding C6 [30,39,40], supporting the fact that this component is problematic when defining the global score. The reliability of the PSQI was also shown by all corrected item-to-total or component-to-total correlations which ranged on average between 0.31 and 0.66, that is, from moderate to high correlations. Only in [38] were the corrected component-to-total correlations low (0.10–0.40). Only six studies tested the reliability of the tool over the time, suggesting that the PSQI was reliable over different periods (from 2 weeks to 14–16 weeks between administrations). The test-retest correlations were high (mean correlations = 0.64) and no difference between administrations was reflected in the mean value scores. The PSQI global score correlated with other sleep measures, such as the ISI, Ford Insomnia Response to Stress Test, and Glasgow Sleep Effort Scale [29,31,38], but not with ESS or Snore, Tired, Observed, Pressure, Body mass index, Age, Neck, Gender (STOP-BANG) [29,31]. The PSQI global score correlated with different tools measuring well-being from different perspectives (e.g., Beck Depression Inventory or Health Survey Short Form 36) with coefficient correlations ranging from −0.40 to 0.72 [30,33,34,37,38]. The correlations between the PSQI score and objective measures of sleep appeared to be more problematic, with a small number of exceptions such as the correlation between global score and Stage 2 latency (r = 0.294), Slow Wave Sleep latency (r = 0.524), Stage 1% (r = 0.327) and Stage 2% (r = −0.349) obtained by PSG [36], between sleep latency measured by both PSG and PSQI (r = 0.225) or between sleep efficiency measured by both PSG and PSQI (−0.331) [32].

In the original paper [21] a cut-off score of 5 was proposed to distinguish between poor and good quality sleepers. This cut-off was used in four papers, supporting known-group validity [31,34,38,39]. Using ROC curve analysis, the reviewed papers did not systematically confirm this cut-off value [25,36,40], but cut-offs greater than 5, such as 6, 7, 8.5 or 11 were more useful in balancing between sensitivity and specificity. Most probably, this differentiation reflects the different populations taken into account and the clinical use of the global PSQI score. The cut-off of 11 was a very severe cut-off used for detecting insomnia patients [25], even if a value equal to 8.5 was a sufficient cut-off for detecting the severity of symptoms in a sample of insomnia patients (211 out of 261) [32]. According to a specific population (university students [40] or sleep-disorder adult patients [29]), a cut-off of 6 or 7 seemed to be able to distinguish insomniacs. Even if further research is needed to clarify the application and the use of specific cut-off points, Table 1 shows that the PSQI had a good construct validity as demonstrated by known-group differences on the basis of proposed cut-off values or other sleep disorder assessments. It is worth noting that the group comparisons were performed according to different cut-off parameters, such as depression level, suggesting the association between poor sleep and different psychological or medical variables [23,26,28,30,31,33,35,36]. Finally, four studies performed regression analyses in order to detect which variable(s) could predict poor sleep; depression, anxiety, and stress were able to predict poor sleep quality [34,38]. As regards gender, females appeared to report a higher PSQI score (and also for C1 and C5 components; [39,41]) even if this result was not confirmed in other studies [27,35]. Only [27] reported the role of age and literacy in predicting sleep quality but these results need further research.

### 3.2. The Sleep-Disorder Scales

Sleep disorders are among the most prevalent complaints in primary medical care and in the general population [4,5,6,7,8,9,10,11,12]. Epidemiological data indicate that insomnia is the most frequent sleep complaint [3]. Insomnia disorder is characterized by difficulty falling asleep, difficulty staying asleep, early morning awakening, and clinical distress or impairments of daily activities [3,13,14,15]. Sleep disturbance and associated daytime symptoms occur at a frequency of three nights or more per week for at least three months. In addition, sleep disorders compromise the sleep-wake cycle and they can affect sleep (hyposomnia) and/or wake (hypersomnia). In this section, we decided to group altogether all scales evaluating more specific alterations of sleep, such as insomnia or different sleep disorders, and complaints of the sleep-wake cycle.

The Athens Insomnia Scale (AIS) [43] is a self-reported questionnaire designed to measure the severity of insomnia based on the diagnostic criteria of the International Classification of Diseases, 10th revision (ICD-10) [44]. There are two versions of the scale: the AIS-8 and the AIS-5. For the eight-item scale, the first five items (assessing difficulty with sleep induction, awakening during the night, early morning awakening, total sleep time, and overall quality of sleep) correspond to criterion A (“complaint of difficulty falling asleep, maintaining sleep or non-refreshing sleep”) for the diagnosis of insomnia according to ICD-10 [44], while the last three items pertain to the consequences of insomnia the next day (problems with sense of well-being, functioning, and sleepiness during the day) according to criterion C of ICD-10 (“the sleep disturbance results in marked personal distress or interference with personal functioning in daily living”). The brief 5-item version is made up of the first five items. In both versions, participants are asked to score each item from 0 (no problem at all) to 3 (very serious problem) if they have experienced any difficulty sleeping at least three times a week during the past month. The total score of the AIS-8 ranges from 0 to 24 while that of the AIS-5 ranges from 0 to 15. Within the selected papers that regard AIS, a great quantity of the studies was performed in Asiatic countries, without modification of the original version. As regards the factor structure of the AIS-8 (Table 2), three studies provided support for unidimensionality [45,46,47] (a mean variance of 67.58% explained; an average range of factor loadings of between 0.54 and 0.85), while three other studies reported a better fit with the two-factor model [48,49,50] with a Nocturnal factor (items 1–5; mean factor loadings ranging between 0.55 and 0.87) and a Daytime Dysfunction factor (items 6–8; mean factor loadings ranging between 0.54 and 0.93), with a mean inter-factor correlation equal to 0.70. Two studies using the AIS-5 found a 1-factor model [45,49] with an average of 57.16% of variance explained, confirming the latent presence of the Nocturnal factor in the full AIS version. The sample size and the type of population assessed (generally trauma patients in studies assessing the two-factor model) may be responsible for these divergent results in the factor analysis. The mean reliability of AIS-8 was 0.86 while that of AIS-5 was 0.84 (Cronbach’s alphas for the two supposed factors were on average higher than 0.80 for the Nocturnal factor and above 0.70 for the Daytime Dysfunction factor). A good internal homogeneity was also demonstrated (mean item-total correlation range 0.56–0.80). With different temporal intervals (from 1 week to about 3 months), the mean ICC of the AIS-8 was 0.78 and that of the AIS-5 was 0.68, suggesting a good test-retest reliability. Finally, both versions of AIS showed convergent/divergent validity (many correlations of a moderate level > 0.30; from −0.53 to +0.85 in range) with different sleep scales, such as PSQI and ISI, and with different psychological variables, such as anxiety or depression [45,46,47,48,49,50], but not with Alcohol Use Disorders Identification Test (AUDIT) or socioeconomic status [46]. The validity of the AIS was also confirmed by known-group differences between patients (psychiatric, insomnia or cancer patients taking opioids) and control or non-insomnia groups (Table 2). Age (older adults) and gender (women) differences were found in the total AIS score as well as for both factors [50]. When a specific cut-off score was proposed, we observed that for AIS-8 values in the range between 5 and 9 [46,48,49] could discriminate between insomnia and non-insomnia groups (Table 2) with a mean sensitivity equal to 80% and a mean specificity equal to 82%, in line with the proposed cut-offs in the original study [44,51]. The different cut-off values reflected the insomnia patients involved in the study and the severity of their insomnia symptoms [46,48,49]. It is worth noting that Enomoto et al. [49] reported a cut-off of 4 for AIS-5.

Similar to the AIS, the Insomnia Severity Index (ISI) measures perceived insomnia severity, focusing on the level of disturbance to the sleep pattern, consequences of insomnia, and the degree of concern and distress related to the sleep problem [52]. Its content corresponds in part to the diagnostic criteria of insomnia outlined in the Diagnostic and Statistical Manual of Mental Disorders (DSM-IV) [53]. The ISI comprises seven items that assess the severity of sleep-onset and sleep maintenance difficulties (both nocturnal and early morning awakening), satisfaction with current sleep pattern, interference with daily functioning, noticeability of impairment attributed to the sleep problem and degree of distress or concern caused by the sleep problem. Each of these items is rated on a five-point Likert scale (0 = not at all; 4 = extremely) and the time interval is “in the past 2 weeks”. Total scores range from 0 to 28 with high scores indicating greater insomnia severity. This tool is available in three different versions: patient (self-administered), significant other, and clinician. All included papers referred to the patient’s version. In the original validation study, different categories were provided: 0–7, no significant insomnia, 8–14, subthreshold insomnia, 15–21, moderate insomnia, and 22–28, severe insomnia [52]. Concerning the factor structure, we found four studies proposing a 1-factor model [47,54,55,56,57] (mean 62.03% of total variance and mean factor loadings ranging from 0.47 to 0.83). It should be noted that Dragioti et al. [54] proposed a four-item version (items 2, 4, 5, 7), especially for patients with chronic pain. However, three studies reported the 2-factor solution [58,59,60] (Severity of sleeping difficulties with items 1–4 or alternatively items 1a, 1b, 1c, and 2; Impact of insomnia with items 5–7 or, alternatively, items 3–5). This solution generally explained 61.80% of variance with mean factor loadings ranging from 0.50 to 0.90 for both factors and mean inter-factor correlation of 0.50 (Table 2). In medical patients, Dieperink et al. [61] considered the 3-factor model, in which Severity of Nighttime Sleep Difficulties (items 1–3 with factor loadings > 0.59), Impact of Insomnia (items 5–7 with factor loadings > 0.72), and Sleep Dissatisfaction/Satisfaction (items 1, 4, 7 with factor loadings > 0.36) correlated with each other with values greater than 0.80. This solution was confirmed in other studies [56,62,63] (Table 2), even if Dieck et al. [56] reported a different composition of factors (F1: items 2, 4, 7; F2: item 1; F3: items 5, 7) and a single correlation between F1 and F3 (0.794). The different numerosity of the samples and the specific characteristics of recruited participants might explain these different results regarding the latent structure of the ISI. The reliability of ISI was good with a mean Cronbach’s alpha of 0.82 and mean corrected item-to-total correlations ranging from 0.47 to 0.66. Importantly, the test-retest reliability was significant in clinical and nonclinical populations [56]. In general, test-retest reliability after 2 weeks was satisfactory (mean ICC = 0.82) [47,56,61] and the ordinal alpha remained above the critical value of 0.70 after a CBT-I treatment [63]. The ISI exhibited significant correlations with several sleep questionnaires such as AIS and PSQI (but low correlation coefficients with ESS) and with different psychological, health, and psychopathological questionnaires. The range of all correlations was between −0.58 and 0.79 (Table 2). Sadeghniiat-Haghighi et al. [59] reported a specific correlation pattern with PSG variables. Indeed, the first three items were associated with PSG variables to a greater extent than the total ISI score was, which correlated only with WASO and SE, even if the correlation coefficients for the first three items were small (<±0.30). In addition, Castronovo et al. [63] reported how the first three items of the ISI were associated with quantitative estimates of sleep parameters (Table 2) obtained from the sleep diaries with moderate correlations, supporting the premise that the first three items have a diagnostic role. As regards validity, the studies demonstrated known-group differences on the basis of different criteria, such as PSQI or depression [47,58,59,61]. In one study [55] women had a higher ISI score than men, and in another study [54] sex was a predictive factor of ISI score, but this gender effect was not systematically confirmed [54,55,61]. Importantly, Castronovo et al. [63] found that ISI was sensitive to change after a specific CBT-I treatment, with a reduction of the higher scores in each item. In our selected papers, only three studies performed a ROC curve analysis; these reported that ISI cut-off was in the range between 9 and 11 with a mean sensitivity of 86% and mean specificity of 80% [47,56,57], depending on the population considered and PSQI cut-off used [47,55,57,59,62]. Taking into account that one study reported an agreement of about 85% between ISI ≥ 8 and PSQI > 5 [62] in the detection of “poor sleepers”, the cut-off values proposed in reviewed articles were in the subthreshold insomnia categories within the 8–14 range [52].

The Mini Sleep Questionnaire (MSQ) [64] is a short questionnaire that can be used to screen sleep disorders in the population and considers complaints regarding both sleep and wake at the same time. The original version was composed of seven items that evaluate symptoms of hypersomnia, and one item on sleep maintenance. Subsequently, three items regarding symptoms of insomnia were added. Thus, the final 10-item version assesses both insomnia and excessive daytime sleepiness. Each item is scored on a seven-point Likert scale ranging from 1 (never) to 7 (always), and takes into account the past seven days. The total sum of scores is divided into four levels of sleep difficulties: 10–24, good sleep quality; 25–27, mild sleep difficulties; 28–30, moderate sleep difficulties; ≥31, severe sleep difficulties [65]. The total score offers an estimate of sleep quality, with higher scores reflecting more serious sleeping problems. However, Natale et al. [66] found two factors explaining about 50% of total variance with loading values higher than 0.50, with the exclusion of item 6 (snoring) (Table 2). The authors labeled Wake (items 4, 5, 8, 9) and Sleep (items 1, 2, 3, 7, 10) dimensions. Thus, the MSQ could be considered a good tool for screening sleep disorders in the population because it consists of two subscales that investigate sleep quality and daytime sleepiness [66]. By contrast, Kim [67] assessed the psychometric properties of MSQ-Insomnia which is composed of four items (difficulty falling asleep, awakening early in the morning and unable to sleep again, taking sleeping pills and tranquilizers, and waking up during the night) with factor loading values higher than 0.50, with the exception of item 3 in the single factor. As reported in Table 2, Natale et al. [66] reported higher Cronbach’s alphas for both factors, with a good internal homogeneity (0.44 for wake factor and 0.37 for sleep factor) while Kim [67] reported a Cronbach’s alpha of 0.69 with an increase of alpha if item 3 was deleted (0.73). The item-total correlation was ≥0.30. Furthermore, the Korean version of MSQ for insomnia subscale [67] correlated with both PSQI (0.22–0.71 range) and Perceived Stress Scale or PSS (0.11–0.31 range), while Natale et al. [66] reported that healthy participants obtained lower scores in the wake dimension of the MSQ in comparison to participants, a result that was compatible with excessive daytime sleepiness; they also found that healthy participants obtained lower scores in the sleep dimension of the MSQ in comparison to participants, compatible with impaired sleep quality (Table 2). Finally, Natale et al. [66] indicated that Wake > 14 and Sleep > 16 were optimal values for detecting hypersomnia and insomnia problems, respectively. Kim [67] evaluated the predictive validity of the MSQ-Insomnia for poor sleepers determined by the diagnostic cut-off on the Korean PSQI score (>8.5 points, [32]), and concluded that it gave a good level of predictive validity.

Finally, in this section we included three tools used for the diagnosis of sleep disorders including insomnia. The first questionnaire was the Jenkins Sleep Scale (JSS) [68]. The JSS is an efficient instrument for the evaluation of the most common symptoms in the general population [68]. JSS is a simple, self-reported, and non-time-consuming scale to be used in daily practice, clinical research, and epidemiologic studies. The questionnaire consists of four items that assess sleep problems over the preceding 4 weeks, with questions regarding trouble falling asleep, trouble staying asleep, frequent awakenings during the night, and subjective feelings of fatigue and sleepiness despite having had a typical night’s rest [69]. The respondents answer the questions using a 6-point Likert-type scale from 0 (not at all) to 5 (22 to 31 days). The total scores range from 0 to 20, and higher scores indicate a greater number of sleep problems [68,69]. In a large representative German sample [69], JSS-4 showed and confirmed the 1-factor solution, explaining a large variance with high factor loading values (Table 2). The JSS-4 proved excellent reliability and it demonstrated good construct validity with regard to mental health, suggesting that sleep problems and psychological distress comprising anxiety, depression, and somatization were moderately related to each other. In addition, the JSS-4 total score was associated with sex, age, education, household income, cohabitation, and employment [69], not only with correlations but also with multivariate analysis. Interestingly, normative data of sleep problems were provided with the percentile rank of each value of the total score provided, allowing comparisons of the JSS-4 scores obtained with different groups of the general population stratified by sex and age. It is worth noting that Tibubos et al. [69] indicated that, in the total sample, a sum score equal to 2 corresponded to 51 in percentile rank, in line with the recommended cut-off of ≥2 to detect sleep disturbances, which corresponds to at least one troubled night per week [68]. The second self-report scale is the Leeds Sleep Evaluation Questionnaire (LSEQ) [70]. The LSEQ comprises ten self-rating 100 mm line analogue questions concerned with sleep and morning behavior and is relatively simple in its use. In the original study [70], factor analysis revealed four independent domains that pertained to sleep latency (or getting to sleep GTS: items 1–3), quality of sleep (QOS: items 4–5), awakening from sleep (AFS: items 6–7), and behavior following wakefulness (BFW: items 8–10). For each item of 100 mm visual analogue, 0 indicated the worst sleep condition and 100 suggested a normal state, and therefore lower scores of the LSEQ indicated poor sleep. In our review, we found an adapted LSEQ that had been administered to Ethiopian university students [71]. In this version (LSEQ-M), not only did the authors modify some items or word expressions (e.g., “usual” was replaced with “normal”), but also the reported score for each item was divided by 10 to determine an individual item score between 0 and 10. Such scores (between 0 and 10) for each item were added to obtain a LSEQ-M global score with a range of 0–100. Interestingly, the authors found 1-, 2-factor and 4-factor models according to different criteria (i.e., eigenvalue > 1, scree plot and cumulative variance > 40). The significant lower values of the LSEQ-M global score as well as those relating to all the items (with the exclusion of item 9) among students with a moderate level of anxiety established the diagnostic known-group validity of the tool. At the cut-off score of 52.6, the sensitivity and specificity of the LSEQ-M were 94% and 80%, respectively (Table 2) [71]. The third self-administered questionnaire is the SLEEP-50 [72], assessing a range of sleep complaints and disorders, including sleep apnea, insomnia, narcolepsy, restless leg syndrome/periodic limb movement disorder, circadian rhythm sleep disorder, sleep walking, and nightmares, in addition to factors which may disrupt sleep, and the impact of sleep complaints on daily functioning. Items are rated on a 4-point Likert scale, from 1 (not at all) to 4 (very much), and each item refers to the past 4 weeks. Items are summed to yield subscale totals and an overall total score, with higher scores indicative of poorer sleep functioning. Spoormaker et al. [72] reported that cut-off scores for each sleep subscale in conjunction with the impact subscale (i.e., greater than or equal to a score of 15) were used to establish the presence of a sleep disorder (i.e., whether symptoms reached a diagnostic threshold). Ricketts et al. [73] added psychometric properties of the SLEEP-50 in two medical conditions (Trichotillomania and Excoriation disorder) in which sleep problems may occur and influence disorder severity. As shown in Table 2, a similar 9-factor model was found for both Trichotillomania and Excoriation Disorder samples, with low factor loadings for item 35 on Factor 7 and item 39 on Factor 8. As far as internal consistency is concerned, values were similar between both groups of patients and comparable to those found in the initial investigation [72]. The internal consistency for the full SLEEP-50 scale was excellent in Trichotillomania and good in Excoriation Disorder, with moderate to strong convergent validity in the association with PSQI global score (from 0.25 to 0.75 and from 0.17 to 0.65 in Trichotillomania and Excoriation Disorder, respectively). The study showed that Trichotillomania and Excoriation Disorder groups exhibited sleep complaints and met a clinical threshold at higher rates (i.e., 63.6% for Trichotillomania and 66.5% for Excoriation Disorder) compared to the control group (39%), suggesting that the SLEEP-50 is a valid self-report tool which may serve to facilitate and standardize screening of multiple sleep complaints among individuals with hair-pulling and skin-picking disorders [73].

**Table 2:** Reported measurement properties of Sleep Disorder Scales: dimensionality, reliability, construct validity, and ROC curve analysis.

			Reliability		Construct Validity			
Study and Abbreviation of Questionnaire	Population	Dimensionality	Internal Consistency	Test-Retest	Convergent/Divergent Validity	Known-Group Validity (Mean Value)	Cut-Off Score or ROC Curve	Version
Gómez-Benito et al., 2011 [45]: AIS-8 and AIS-5	A total of 323 individuals (227 females); mean age of 30.29 years: 167 students (138 females; mean age of 20.50 years), 77 psychiatric outpatients (38 females; mean age of 40.16 years) and 79 community sample (56 females; mean age of 43.42 years)	AIS-8: 1-factor model (51.50% of variance explained) and factor loading ranging from 0.488 to 0.858. AIS-5: 1-factor model (54.33% of variance explained) and factor loading ranging from 0.614 to 0.818	Cronbach’s alpha for AIS-8 was 0.86. Cronbach’s alpha for AIS-5 was 0.79.For AIS-8 Cronbach’s alphas for undergraduates, patients and the general population were 0.81, 0.89, and 0.86, respectively. For AIS-5 Cronbach’s alpha for undergraduates, patients and the general population were 0.66, 0.83, and 0.78, respectively	A 1-month test-retest for AIS-8 (ICC = 0.75) and for AIS-5 (ICC = 0.64)	AIS-8 and AIS-5 correlated with BDI (0.53 and 0.46, respectively), with BAI (0.49 and 0.42 respectively) and with GHQ-12 (0.54 and 0.44 respectively)	For AIS-8: students (4.83) < community sample (6.20) < psychiatric patients (8.12). For AIS-5: students (2.82) < community sample (4.22) or psychiatric patients (5.22)		Spanish
Okajima et al., 2013 [48]: AIS-J	A total of 640 individuals (371 females); mean age of 48.8 years: 477 outpatients with chronic insomnia (253 women; mean age of 47.9 years) and 163 individuals who scored less than 6 points on PSQI-J (54 women; mean age of 51.3 years)	A 2-factor structure: Nocturnal Sleep Problems (AIS-J-nocturnal, items 1–5; factor loading 0.33–0.87) and Daytime Dysfunction (AIS-J-daytime, items 6–8; factor loading 0.45–0.94).Inter-factor correlation was 0.62	Cronbach’s alpha for total score was 0.88 and for F1 and F2 it was 0.85 and 0.78, respectively		There was a correlation between AIS-J and PSQI-J (0.81) and between AIS-J and ISI-J (0.85). In patients with insomnia there were correlations between AIS-J and PSQI-J (0.57) and between AIS-J and ISI-J (0.58) but no significant correlations were found in patients with depression or those with anxiety disorder (rs n.a.)	Insomnia group with higher scores than control group for AIS-J (11.81 vs. 2.64), Nocturnal (8.12 vs. 1.63) and Daytime (3.65 vs. 1.01) factors. Insomnia, depression and anxiety disorder groups (above 11) > healthy controls (less than 3). Same results for Nocturnal factor (above 6 for patients and below 2 for controls). For Daytime factors, depression and anxiety disorder groups (above 4) had higher scores than primary insomnia (about 3) and controls (about 1)	AIS-J: ROC curve for insomnia (primary and secondary) with AUC = 0.96 for cut-off of 5.5 and sensitivity = 92% and specificity = 93% (LR+ = 13.62 and LR− = 0.09. AIS-J-nocturnal: ROC curve for insomnia (primary and secondary) with AUC = 0.97 for cut-off of 3.5 and sensitivity = 93% and specificity = 94% (LR+ = 16.73 and LR− = 0.07). For primary insomnia AIS-J > 5.5 with AUC = 0.97, sensitivity = 93%, specificity = 93%, LR+ = 13.78, LR− = 0.08; AIS-J—nocturnal > 3.5 with AUC = 0.97, sensitivity = 94%, specificity = 94%, LR+ = 16.88, LR− = 0.07	Japanese
Jeong et al., 2015 [46]: AIS-8	A total of 221 firefighters and rescue workers (14 women); mean age of 40.3 years	A 1-factor structure (95.73% of variance explained) with factor loading of each item ranging from 0.51 to 0.82	Cronbach’s alpha of 0.88. Mean item-total correlation coefficient was 0.73 (0.56–0.84 range)	A 1 week test-retest with ICC for total score equal to 0.94 (0.58–0.95 range)	AIS-8 correlated with PSQI (0.82), ISI (0.85), ESS (0.29), SF-36 mental component summary (−0.53), SF-36 physical component summary (−0.37), but not with AUDIT-C (0.10) or socioeconomic status (0.01)	Based on Structured Clinical Interview for the DSM-IV-TR and a structured clinical interview for insomnia, participants were divided into non-insomnia, participants with insomnia symptoms (group 1), individuals with disturbed daily functioning (group 2) and those in group 2 who had symptoms even during off-duty periods.Non-insomnia group (4.1) < group 1 (9.3) < group 2 (10.1)	ROC curve for group 1 with cut-off score AIS-8 = 6, AUC = 0.87, sensitivity = 87%, specificity = 72%, LR+ = 3.07, LR− = 0.18; ROC curve for group 2 with cut-off score AIS-8 = 8, AUC = 0.84, sensitivity = 73%, specificity = 79%, LR+ = 3.50, LR− = 0.35; ROC curve for group 3 with cut-off score AIS-8 = 9, AUC = 0.85, sensitivity = 71%, specificity = 84%, LR+ = 4.45, LR− = 0.34	Korean
Enomoto et al., 2018 [49]: AIS-8 and AIS-5	A total of 144 outpatients with a history of pain (86 females); mean age of 53.3 years	AIS-8: 2-factor model without item 8 with poor factor loading: Nocturnal Sleep Problem (items 1–5) and Daytime Dysfunction (6–7). AIS-5: 1-factor (Nocturnal Sleep Problem) model with a covariation between item 1 and item 5 (0.30) and factor loading > 0.60	Cronbach’s alpha for AIS-8 was 0.87 and for AIS-5 was 0.89. For nocturnal sleep problems the Cronbach’s alpha was 0.89 and for daytime dysfunction was 0.66	An 87.4 day test-retest with overall ICC of 0.64 for AIS-8, 0.72 for the AIS-5 and nocturnal sleep problems and 0.54 for daytime dysfunction	AIS-8 correlated with NRS (0.36), PDAS (0.46), HADS-anxiety (0.54), HADS-depression (0.64), PCS-total (0.36), PCS-rumination (0.23), PCS-magnification (0.37), PCS-helplessness (0.35), PSEQ (−0.47). AIS-5 correlated with NRS (0.35), PDAS (0.37), HADS-anxiety (0.42), HADS-depression (0.52), PCS-total (0.26), PCS-rumination (0.17), PCS-magnification (0.27), PCS-helplessness (0.24), PSEQ (−0.35)	Based on the semi-structured interview data, participants were divided into an insomnia group and non-insomnia group. Insomnia group (AIS-8 = 11.4; AIS-5 = 7.1) > non-insomnia group (AIS-8 = 5.2; AIS-5 = 2.7)	ROC curve to detect insomnia with AIS-8 = 8, AUC = 0.82, sensitivity = 72%, specificity = 85%. ROC curve to detect insomnia with AIS-5 = 4, AUC = 0.82, sensitivity = 78%, specificity = 70%	Japanese
Iwasa et al., 2018 [50]: AIS-SJ	A total of 50,547 community dwellers who lived in the evacuation zone designated by the government for Fukushima Dalichi NPP Incident (27.669 women); mean age of 52.9 years	A 2-factor model: F1 or Nocturnal (items 1–5; factor loading from 0.71 to 0.87) and F2 or Daytime (items 6–8; factor loading from 0.62 to 0.91). Inter-factor correlation was 0.77	Cronbach’s alpha for all 8 items was 0.81. Cronbach’s alpha for F1 was 0.80 and for F2 was 0.76		Correlations appeared between total AIS-SJ score and K6 scale (0.60), PCL-S (0.60), mental illness (0.36), self-rated health (0.51), experiencing tsunami (0.10), experiencing NPP incident (0.18), bereavement (0.17), housing damage (0.13) and losing job (0.15). There was the same correlation pattern for F1 (0.10–0.54 range) and for F2 (0.08–0.56 range)	Young men (2.76) had similar AIS-SJ score to that of old men (2.82). Older women (3.49) had higher AIS-SJ score than young women (3.19). Women (3.27) had higher AIS-SJ score than men (2.80). Older adults (2.06) had higher Nocturnal score than younger adults (1.86). Women (2.12) had higher Nocturnal score than men (1.80). Younger adults (1.12) had higher Daytime score than older adults (1.09). Women (1.21) had higher Daytime score than men (1.00)		Japanese
Lin et al., 2020 [47]: AIS-8 and ISI	A total of 573 patients with cancer at stage III or IV (247 females); mean age of 61.3 years	AIS-8: 1-factor structure (adequate average variance extracted = 0.56) with factor loadings from 0.61 to 0.87 and Rasch-derived infit (0.81 to 1.17) and outfit (0.79 to 1.14) mean square fitted the underlying construct; no substantial DIF was found across the sex (DIF contrast = −0.43 to 0.43) or insomnia condition (DIF contrast = −0.23 to 0.19). ISI: 1-factor model (adequate average variance extracted = 0.54) with factor loadings from 0.61 to 0.81 and Rasch-derived infit (0.72 to 1.14) and outfit (0.76 to 1.11) mean square fitted the underlying construct; no substantial DIF was found across the sex (DIF contrast = −0.12 to 0.48) or insomnia condition (DIF contrast = −0.19 to 0.33)	AIS-8: satisfactory internal consistency (ω = 0.88), high composite reliability (0.91), low standard error of measurement (2.57), corrected item-total correlations from 0.56 to 0.76, separation reliability (0.88 and 0.84) and separation indices (2.75 and 2.30) were acceptable. ISI: satisfactory internal consistency (ω = 0.79), high composite reliability (0.89), low standard error of measurement (2.00), corrected item-total correlations from 0.43 to 0.67, separation reliability (0.98 and 0.78) and separation indices (7.20 and 2.71) were acceptable	A 2-week test-retest reliability for AIS-8 was satisfactory (0.72 to 0.82) and ICC = 0.82. 2-week test-retest reliability for ISI was satisfactory (0.72 to 0.82) and ICC = 0.79	AIS-8 and ISI were mutually correlated (0.64). AIS-8 was correlated with ESAS (0.38), HADS-anxiety (0.58), HADS-depression (0.56), KPSS (−0.50), GHQ-12 (0.61), ESS (0.62) and PSQI (0.55).ISI correlated with ESAS (0.41), HADS-anxiety (0.53), HADS-depression (0.62), KPSS (−0.41), GHQ-12 (0.54), ESS (0.64), and PSQI (0.58)	AIS-8 and ISI: patients who took opioids had the highest AIS scores (9.48 and 10.33) followed by those taking non-opioid analgesics (7.13 and 7.60) and those taking other medications (7.29 and 6.41). AIS < 7 (insomniacs) vs. AIS > 7 (non-insomniacs): difference for actigraph data of TST, SE, bedtime, wake time, SOL, and WASO. ISI < 9 (insomniacs) vs. ISI > 9 (non insomniacs): difference for actigraph data of TST, SE, bedtime, wake time, SOL, and WASO	ROC curve with cut-off score of AIS-8 = 7 for detecting insomnia with AUC = 0.86, sensitivity = 86% and specificity = 81%. ROC curve with cut-off score of ISI = 9 for detecting insomnia with AUC = 0.82, sensitivity = 86% and specificity = 83%. AIS-8 and ISI cut-offs were consistent with psychiatric diagnosis based on DSM-IV	Persian
Yu, 2010 [58]: ISI-C	A total of 585 Chinese community-dwelling older people (474 females); mean age of 74.3 years	A 2-factor model (61.40% of the total variance) with factor loadings ranging from 0.56 to 0.85: F1 (items 1–4) assessing severity of sleeping difficulties; F2 (items 5–7) assessing daytime interference and distress associated with insomnia as well as how noticeable the sleeping problem was	Cronbach’s alpha of 0.81. Corrected item-to-total correlation for the items was in the range of 0.33–0.67.Cronbach’s alpha for F1 = 0.788. Cronbach’s alpha for F2 = 0.640		ISI-C correlated with CPSQI (0.686) and with sleep efficiency derived from CPSQI item scores (−0.583)	Depressed adults reported by GDS had higher scores on the ISI-C (12.61) than those without this problem (9.19). Poorer sleepers defined by the CPSQI cut-off point ≥ 5 had a higher ISI-C score than those of normal sleepers [mean not reported]		Chinese
Fernandez-Mendoza et al., 2012 [62]: ISI	A total of 500 adults from the general population (307 females); mean age of 39.13 years	A 3-factor model with Impact of Insomnia (items 5–7), Sleep Dissatisfaction (items 1, 4, 7) and Night-time Sleep Difficulties (items 1–3).Mean inter-item correlations were 0.35 (night-time sleep difficulties), 0.59 (impact of insomnia) and 0.50 (sleep dissatisfaction). Inter-factor correlations were found between Impact of Insomnia and Sleep Dissatisfaction (0.61), between Sleep Dissatisfaction and Night-time Sleep Difficulties (0.83) and between Impact of Insomnia and Night-time Sleep Difficulties (0.48)	Cronbach’s alpha was 0.82. Cronbach’s alpha of factors was 0.60 (for night-time sleep difficulties), 0.81 (for impact of insomnia) and 0.75 (for sleep dissatisfaction). Corrected item-to-total correlation for the items ranged from 0.47 to 0.71		ISI correlated with PSQI (0.68), ESS (0.18), POMS-fatigue (0.40), POMS-depression (0.34) and POMS-anxiety (0.38). Impact of insomnia correlated with PSQI (0.49), ESS (0.26), POMS-fatigue (0.45), POMS-depression (0.38) and POMS-anxiety (0.41). Sleep dissatisfaction correlated with PSQI (0.68), ESS (0.11), POMS-fatigue (0.34), POMS-depression (0.27) and POMS-anxiety (0.33). Sleep difficulties correlated with PSQI (0.62), POMS-fatigue (0.23), POMS-depression (0.22) and POMS-anxiety (0.23), but not ESS (0.04)		85% of subjects classified as insomniacs in the ISI total score ≥8 were classified as poor sleepers in the PSQI total score > 5 and 33% of non-insomniacs were classified as poor sleepers	Spanish
Sadeghniiat-Haghighi et al., 2014 [59]: ISI-P	A total of 1037 patients referred to a sleep disorder clinic (301 females; mean age of 45.4 years) and 50 hospital staff (31 females; mean age of 32.1 years)	A 2-factor model (60.58% of variance observed): F1: item 1a, item 1b, item 1c and item 2 (factor loading ranged from 0.57 to 0.77); F2: items 3–5 (factor loading ranged from 0.64 to 0.83)	Cronbach’s alpha in patients was 0.78. Corrected item-total correlations ranged from 0.35 to 0.63		ISI-P correlated with ESS (0.12), BDI (0.42) and PSQI (0.74) ISI total score correlated with PSG variables such as WASO (0.12) and SE (−0.13). Item 1a correlated with WASO (0.17), EMA (0.14), TST (−0.22), TWT (0.19) and SE (−0.24). Item 1b correlated with WASO (0.12), TST (−0.12) and SE (−0.14). Item 1c correlated with SOL (−0.12), WAS (0.12), EMA (0.17), and SE (−0.10). Item 2 correlated with SE (−0.10). Item 1a correlated with C2-PSQI (0.61), item 1b correlated with C5-PSQI (0.18), item 1c correlated with C5-PSQI (0.13), item 2 correlated with C1-PSQI (0.41), and item 3 correlated with C7-PSQI (0.43)	Patient group (15.90) > control group (10.10)		Persian
Dragioti et al., 2015 [54]: ISI-4	A total of 836 patients with chronic pain (269 men with mean age of 50 years and 567 women with mean age of 45 years)	And 1-factor model (63.1% of variance explained) with factor loadings ranging from 0.598 to 0.880. In 109 men the 1-factor solution was confirmed (66.8% of variance explained with factor loadings from 0.591 to 0.905).In 225 women the 1-factor solution was confirmed (62.4% of variance explained with factor loadings from 0.597 to 0.880). In 502 patients the 1-factor solution was found with only 4 items (items 2, 4, 5 and 7: factor loadings from 0.72 to 0.88) and no sex difference was found	Cronbach’s alpha of ISI-4 was 0.88. Component-to-total score correlations were high (0.65–0.80). Inter-component analysis revealed correlations between items from 0.53 to 0.75		ISI-4 correlated with HADS-anxiety (0.37), HADS-depression (0.35), quality of sleep (0.65) and mental dimension of quality of life (−0.35). No correlation with age (*p* > 0.05)	No gender difference (men = 10.22 vs. women = 9.80). Multiple linear regression analysis showed that both sex and pain duration affected the score of the ISI-4 whereas pain intensity was associated with the ISI-4 score		Swedish
Castronovo et al., 2016 [63]: ISI	A total of 272 consecutive patients (165 females) with insomnia diagnosis and enrolled in a CBT-I; mean age of 41.36 years	A 3-factor model with Impact (items 3–5), Satisfaction (items 1a, 2, 5) and Severity (items 1a, 1b, 1c). There were inter-factor correlations between Impact and Satisfaction (0.45), between Impact and Severity (0.25) and between Satisfaction and Severity (0.76). Factor loadings in absolute value ranged from 0.33 to 0.99	Ordinal alpha was 0.75 with an increase to 0.76 with the first item removed. The corrected item-to-total correlation for the items ranged from 0.49 to 0.74	After CBI-I treatment the ordinal alpha was 0.73	Correlations between the severity ratings obtained in the first three items of the ISI with corresponding quantitative estimates of SOL (0.44 ISI1a), WASO (0.33 ISI1b), NAWK (0.28 ISI1b), EMA (0.44 ISI 1c) obtained from the sleep diaries, the total ISI score and SE (−0.28) variable from the sleep diary correlated with the Impact scale and SOL (0.14; but not with NAWK, WASO, EMA, SE), Satisfaction scale and SOL (0.36), WASO (0.19), EMA (0.19), SE (−0.26; but not with NAWK) and between Severity scale and SOL (0.21), NAWK (0.20), WASO (0.32), EMA (0.45), SE (−0.39)	Follow-up evaluations from baseline and follow-up after a CBT-I treatment: percentage of responses 3, 4, and 5 decreased, indicating a general improvement in patients’ conditions. Total score of the ISI and scores obtained in each scale were lower after CBT-I		Italian
Gerber et al., 2016 [55]: ISI	Study 2: 862 students (639 women) with mean age of 24.7 years. Study 3: 533 employees of the police force and emergency response service corps (122 women) with mean age of 41.2 years	Studies 2 and 3: 1-factor solution for men and women with factor loadings from 0.11 to 0.90 (males) and from 0.11 to 0.89 (females). Item 5 with factor loadings of 0.31 and 0.29 for Study 2 and of 0.26 and 0.26 for Study 3. Item 6 with factor loadings of 0.11 and 0.11 for Study 2 and of 0.13 and 0.13 for Study 3	Study 2: Cronbach’s alpha was 0.77 in the total sample, 0.78 for men and 0.76 for women. Inter-item correlations were above the critical value of 0.20 (with the exclusion of item 6). Item-total correlations were found for men and women (with mean correlations of 0.51 for men and 0.49 for women) Study 3: Cronbach’s alpha was 0.81 in the total sample, 0.81 for men and 0.82 for women Inter-item correlations exceeded the critical value of 0.20 (with few cases for item 6). Item-total correlations were on average correlations of 0.55 for men and 0.56 for women		Study 2: for males correlations were found between total ISI scores and each item and PSQI with 0.14–0.55 range with few exceptions (below 0.10). Total ISI correlated with all sleep variables of PSQI (range −0.19 and 0.54) with the exception of sleep duration. There were correlations between all items and total scores (0.31–0.50 range; but not item 6) with Depression scale.The same pattern appeared for females (0.13–0.69). ISI correlated with the Depression scale (0.19–0.51 range). Study 3: for males there were correlations between ISI and each item with PSQI (0.11–0.69 range with few exception with r < 0.10). ISI and each item correlated with SF-12 (from −0.20 to −0.46). The same pattern was evident in females with PSQI (0.18–0.75 range) and with SF-12 (from −0.22 to −0.45 with the exception of item 6)	Study 2: women (6.80) > men (5.91) for total ISI score. There were also gender differences for item 1 and item 2 but not for the other items.Study 3: no gender difference was evident for the total ISI score (women: 7.00 and men: 6.97) and there were no differences for any item	Cut-off: 0–7 no clinically significant insomnia,8–14 sub-threshold insomnia, 15–21 clinical insomnia (moderate severity) 22–28 clinical insomnia (severe)	German
Dieck et al., 2018 [56]: ISI	A total of 700 participants (573 females); mean age of 32.16 years	A 3-factor model: F1 (items 2, 4, 7), F2 (items 1), F3 (items 5–7). Item 3 was not assigned to one factor. Correlations between F1 and F3 (0.744), but not for F1 and F2 (0.209) or between F2 and F3 (0.180). 1-factor model estimated with the quartimin rotation method and weighted least squares method: factor loadings from 0.409 (item 3) to 0.901 (item 7)	Cronbach’s alpha of the ISI was 0.83 and the item-total correlation ranged from 0.36 to 0.77	A 2-week test-retest for total score of the ISI was 0.77, and the individual items ranged from 0.51 to 0.73 with only item 3 (0.54) and item 6 (0.51 showed weak test-retest. Test-retest with PSQI ≤5 (0.31–0.58) and PSQI > 5 (0.42–0.78). Test-retest with PSQI > 5 and BDI-II < 20 (0.35–0.74)	Correlations between ISI and PSQI (0.79, 0.61, 0.77), between ISI and BDI-II including sleep item (0.55, 0.37, 0.66), between ISI and BDI-II excluding sleep item (0.56, 0.36, 0.64) and between ISI and SRS (0.36)		Based on PSQI value ≤5 ROC curve with cut-off ISI > 10 with AUC = 0.94, sensitivity = 91% and specificity = 84% (LR+ = 5.86; LR− = 0.10). Based on BDI-II < 20 ROC curve with cut-off score ISI > 10 with sensitivity = 87.28%, specificity = 84.72% (LR+ = 5.71, LR− = 0.15)	German
Kaufmann et al., 2019 [57]: ISI	A total of 83 Operation Enduring Freedom/Operation Iraqi Freedom/Operation New Dawn veterans with a history of TBI (11 females); mean age of 32.5 years	1-factor model (69.0% of variance explained) with total eigenvalues = 4.83 and factor loadings > 0.70	Cronbach’s alpha was 0.92		ISI total score was correlated with NSI (0.76) and BDI-II (0.56) sleep item, the PSQI global score (0.76) and with the ESS total score (0.32). The ISI was correlated with the PSQI individual component score (ranging from 0.324 to 0.791, with the exclusion of C6 with 0.226). ISI total score correlated with BAI (0.450), PCL-M total with sleep item excluded (0.513) and BDI-II with sleep item excluded (0.476)		Categorical scores:0–7 as no insomnia 8–14 as sub-threshold insomnia 15–21 as moderate insomnia 22–28 as severe insomnia. Cut-off of ISI > 11 with 67.5% were classified as having clinical insomnia. Based on PSQI cut-off score of > 8 to indicate elevated insomnia symptoms, ROC curve with AUC = 0.87, sensitivity = 81% and specificity = 71% with cut-off ISI > 11.5	English (American)
Dieperink et al., 2020 [61]: ISI-DK	A total of 249 patients with a medical condition (158 females); mean age of 58.2 years	A 2-factor model with Severity factor (items 1–4; factor loadings from 0.57 to 0.88) and Impact factor (items 5–7; factor loadings from 0.73 to 0.90) and correlation between factors (0.88). 3-factor model with Severity factor (items 1–3; factor loadings from 0.59 to 0.92), Impact factor (items 5–7; factor loadings from 0.72 to 1.30) and Dissatisfaction factor (items 1, 4, 7; factor loadings from −0.36 to 0.85) and inter-factor correlations between severity and dissatisfaction (0.94), between severity and impact (0.81) and between dissatisfaction and impact (0.95)	Cronbach’s alpha was 0.90 with item-total correlation interval between 0.52–0.80 and a mean value of 0.71.When item 3 was deleted the Cronbach’s alpha increased to 0.91. For the 2-factor model, the Cronbach’s alpha of Severity factor was 0.83 and that of Impact factor was 0.88. For the 3-factor model the Cronbach’s alpha of Severity factor was 0.75, that of Dissatisfaction factor was 0.81 and that of Impact factor was 0.88	17.1 days test-retest with ICC = 0.90, with SEM = 2.52, SDC = 6.99 and LoA = 0.05		No gender difference (male = 9.36 vs. female = 10.74). Responders ≥70 years old (8.85) had lower ISI-DK scores compared to younger responders (10.65).Responders with EQ VAS score < 83.7 (11.21) had a higher ISI-DK score compared to responders with a higher EQ VAS score (7.18). Responders with anxiety/depression (12.50) had a higher IS-DK score compared to responders with no problem (8.23).Responders with pain/discomfort problems (11.21) had higher ISI-DK scores compared to responders with no problem (7.44)	Using the insomnia cut-offs 25.4% had moderate insomnia (15–21) and 2.4% had severe insomnia (22–28)	Danish
Manzar et al., 2020 [60]: ISI	A total of 406 substance-using community dwelling adults (54 females); mean age of 27 years	A 2-factor model with the incorporation of modification indices to covary the error terms (cumulative variance explained = 63.41%): F1 (items 1–3; factor loading from 0.67 to 0.76) and F2 (items 4–7; factor loading from 0.51 to 0.80). Inter-factor correlation was 0.52	Cronbach’s of F1 was 0.68 and of F2 was 0.78.The item-total correlations of the ISI were 0.47–0.72. Inter-item (homogeneity) correlations ranged from 0.11 to 0.57 with the exception of the correlation between item 1 and item 7		All of the item scores of the ISI, both factor scores and the ISI total score, correlated with the meta-cognition total score (0.16–0.44 range) and its factor scores: meta-memory (0.19–0.35 range) and meta-concentrations (0.10–0.44 range)			Ethiopian
Natale et al., 2014 [66]: MSQ	A total of 1830 university students and their parents/grandparents (1073 women); mean age of 35.70 years	A 2-factor model (49.8% of variance explained): Wake dimension (items 4, 5, 8, 9; factor loadings from 0.52 to 0.83) and Sleep dimension (items 1–3, 7, 10; factor loadings from 0.51 to 0.75). Only item 6 (snoring) had a loading value of 0.39 and it was not loaded on any dimension. This 9-item solution was a better model in comparison to the 10-item solution	Cronbach’s alpha for the MSQ was 0.77. The average inter-item correlation (homogeneity) was 0.26, ranging between −0.01 and 0.58. Cronbach’s alpha of wake dimension was 0.75 and that of homogeneity 0.44. Cronbach’s alpha of sleep dimension was 0.75 and homogeneity amounted to 0.37			Based on SDQ, healthy participants obtained lower scores in the wake dimension (11.92) in comparison to participants compatible with EDS (17.27). Healthy participants obtained lower scores in the sleep dimension (12.48) in comparison to participants compatible with impaired sleep quality (18.75)	ROC curve with cut-off value for wake dimension > 14 with AUC = 0.83, sensitivity = 78%, specificity = 74%, PPV = 0.29 and NPV = 0.96. ROC curve with cut-off value for sleep dimension > 16 with AUC = 0.82, sensitivity = 73%, specificity = 80%, PPV = 0.40, and NPV = 0.94	Italian
Kim, 2017 [67]: MSQ-Insomnia	A total of 470 students from six nursing colleges in South Korea (437 females); mean age of 21.40 years	A 1-factor model (56.0% of the variance explained): MSQ-Insomnia with 4 items (items 1–4) loading from 0.33 (item 3: taking sleeping pills and tranquilizers) to 0.89 (item 2: awakening early in the morning and unable to sleep again)	Cronbach’s alpha of MSQ-Insomnia was 0.69. Item-total correlations ranged from 0.30 (item 3) to 0.68 (item 2). Cronbach’s alpha increased to 0.73 if item 3 was deleted	Test-retest with ICC = 0.84. No difference in MSQ-Insomnia score between baseline and retest	MSQ-Insomnia correlated with PSQI (0.69), as well as each item of MSQ-Insomnia correlated with PSQI (item1: 0.71, item 2: 0.52, item 3: 0.22, item 4: 0.42). MSQ-Insomnia correlated with PSS (0.31) as well as each item of MSQ-Insomnia correlated with PSS (item 1: 0.26, item 2: 0.25, item 3: 0.11, item 4: 0.24)		Based on the PSQI cut-off score of 8.5, 98 students were classified as poor sleepers. The MSQ-Insomnia had a good level of predictive validity (AUC = 0.85) to predict poor sleepers	Korean
Tibubos et al., 2020 [69]: JSS-4	A total of 2515 representative individuals of the German population (1350 females); mean age of 50.53 years	A 1-factor model (eigenvalue of 3.10) accounting for 77.5% of total variance. Factor loadings ranged between 0.83 and 0.93. 1-factor solution confirmed with CFA (standardized factor loadings ranged between 0.71 and 0.95)	Cronbach’s alpha of 0.90 and Mc Donald’s omega of 0.90. Corrected item-total correlations ranged from 0.69 (item 4) to 0.86 (item 3) in total sample (range 0.67–0.86 in men and 0.70–0.86 in women)		Correlations were present between JSS-4 total and sex (0.10), age (0.28), household income (−0.19), BSI-18 total score (0.51), BIS-18 anxiety (0.42), BSI-18 depression (0.41), and BSI-18 somatic symptom load (0.45).There was the same correlation pattern for item 1 (from −0.17 to 0.43), item 2 (from −0.17 to 0.43), item 3 (from −0.20 to 0.44), and item 4 (from −0.15 to 0.49)	Women (4.23) showed a higher JSS-4 total than men (3.37). There was a linear increase of JSS-4 total score from 14–20 years (2.03) to ≥71 years (5.92).Not living with a partner (4.31) induced a higher JSS-4 score than living with a partner (3.47). JSS-4 total decreased as education level increased (from less education (≤8 years: 4.52 to university students: 1.74). Being retired (5.43) or unemployed (4.67) determined a higher JSS-4 total score than being a student (1.96) or worker (2.87). There was a linear decrease of JSS-4 total score from <€1000 household income (5.78) to ≥€2500 (2.81). Multivariate analysis showed that sleep problems were moderately linked (0.46) with global psychological distress in the specified model. As expected, female (β = 0.05 and 0.08, respectively), older (β = 0.24 and β = 0.05, respectively), and low income (β = −0.18 and β = −0.21, respectively) individuals were more likely to report sleep problems and psychological distress	According to the significant differences in JSS-4 scores between age groups and both sexes, norm values for the total sample as well as for each combination of age and sex separately were provided in percentile ranks: JSS-4 score = 0 corresponded to 33 percentile in total sample; JSS-4 score = 20 corresponded to 100 percentile in total sample	German
Manzar et al., 2018 [71]: LSEQ-M	A total of 424 Ethiopian university students (74 females); mean age of 21.87 years	1-factor model with cumulative variance rule > 40%. 2-factor model with eigenvalue > 1 and scree plot: F1 (items getting to sleep 1–3, quality of sleep 1–2) and F2 (items awake following sleep 1–2, behaviour following wakening 1–2). The behaviour following wakening item 3 did not load in any factor. In confirmatory analysis no model had the best fit values even if the original 4-factor correlated model performed best in some values but not all	Cronbach’s alpha was 0.84. Item-total correlations ranged from 0.60 to 0.69			Based on GAD-7 score, normal participants had higher scores than those with moderate anxiety for LSEQ-M total score (66.92 vs. 52.65, respectively) and for all items with the exception of behavior, following wakening item 2	ROC curve with LSEQ-M score cut-off value of 52.6, with AUC = 0.95, sensitivity = 94% and specificity = 80%	Ethiopian (Mizan)
Ricketts et al., 2019 [73]: SLEEP-50	A total of 234 patients with Trichotillomania (227 females; mean age of 32.30 years), 170 patients with Excoriation disorder (162 females; mean age of 36.40 years) and 146 healthy adults (145 females; mean age of 38.60 years)	Trichotillomania: 9-factor model with Sleep Apnea (items 1–8; factor loadings range 0.41–0.89), Insomnia (items 9–16; factor loadings range 0.50–0.90), Narcolepsy (items 17–21; factor loadings 0.52–0.69), Restless Legs/Periodic Limb Movement Disorder (items 22–28; factor loadings range 0.49–0.95), Circadian Rhythm Sleep Disorder (items 26–28; factor loadings range 0.49–0.73), Sleepwalking (items 29–31; factor loadings range 0.56–0.84), Nightmares (items 32–36; factor loadings range 0.39–0.65 with poor loading for item 35); Factors influencing sleep (items 37–43; factor loadings range 0.37–0.80 with poor loading for item 39), Impact of sleep complaints on daily functioning (items 44–50; factor loading range 0.59–0.94). Inter-factor correlations ranged from −0.21 to 0.76). Excoriation disorder: 9-factor model with Sleep Apnea (items 1–8; factor loadings range 0.32–0.80), Insomnia (items 9–16; factor loadings range 0.63–0.83), Narcolepsy (items 17–21; factor loadings 0.54–0.83), Restless Legs/Periodic Limb Movement Disorder (items 22–28; factor loadings range 0.64–0.92), Circadian Rhythm Sleep Disorder (items 26–28; factor loadings range 0.44–0.70), Sleepwalking (items 29–31; factor loadings range 0.59–1.12), Nightmares (items 32–36; factor loadings range 0.36–0.58 with poor loading for item 35); Factors influencing sleep (items 37–43; factor loadings range 0.17–0.87 with poor loading for item 39), Impact of sleep complaints on daily functioning (items 44–50; factor loading range 0.69–0.86). Inter-factor correlations ranged from −0.81 to 0.81)	Trichotillomania:Cronbach’s alpha for the full scale was 0.91. Alphas for the individual subscales were: Sleep apnea (0.70), insomnia (0.85), narcolepsy (0.47), restless legs/PLMD (0.74), circadian rhythm sleep disorder (0.55), sleepwalking (0.39), nightmares (0.65), factors influencing sleep (0.56), and impact of sleep complaints on daily functioning (0.87). Excoriation disorder: Cronbach’s alpha for the full scale was 0.89. Alphas for the individual subscales were: Sleep apnea (0.67), insomnia (0.85), narcolepsy (0.63), restless legs/PLMD (0.74), circadian rhythm sleep disorder (0.47), sleepwalking (0.66), nightmares (0.66), factors influencing sleep (0.41), and impact of sleep complaints on daily functioning (0.86)		Trichotillomania: there were correlations between SLEEP-50 overall complaints (0.71) and SLEEP-50 impact (0.63) subscales and the global PSQI. Similarly, correlations were found between SLEEP-50 overall complaints (0.62) and SLEEP-50 impact (0.60) subscales and PSQI overall sleep quality subscale (ranging from 0.16 to 0.75). Excoriation disorder: correlations were present between SLEEP-50 overall complaints (0.58) and impact (0.56) subscales and the global PSQI. Similarly, correlations were found between SLEEP-50 overall complaints (0.44) and impact (0.60) subscales and PSQI overall sleep quality subscales (ranging from 0.10 to 0.65)	There were differences between groups based on SLEEP-50 cut-off scores ( ≥ 15) for sleep apnea (trichotillomania 17.1%, excoriation 19.7% and control 6.5%), narcolepsy (trichotillomania 32.9%, excoriation 36.3% and control 16.1%), restless leg/periodic limb movement disorder (trichotillomania 19.7%, excoriation 24.4% and control 11.4%), circadian rhythm sleep disorder (trichotillomania 13.8%, excoriation 12.1% and control 5.1%) and with more than one sleep disorder (trichotillomania 63.6%, excoriation 66.5% and control 39.0%)		English (American)

Abbreviations: BDI: Beck’s Depression Inventory; BAI: Beck’s Anxiety Inventory; GHQ-12: General Health Questionnaire-12; PSQI-J: Japanese version of Pittsburgh Sleep Quality Inventory; ISI-J: Japanese version of Insomnia Severity Index; ROC: receiver-operator curve; AUC: area under the curve; LR+: positive likelihood ratio; LR−: negative likelihood ratio; ESS: Epworth Sleepiness Scale; SF-36: Short-Form 36-item Health Survey; AUDIT-C: Alcohol Use Disorder Identification Test-Consumption; DSM-IV-TR: Diagnostic and Statistical Manual of Mental Disorders-IV-Text Revision; NRS: Numerical Rating Scale; PDAS: Pain Disability Assessment Scale; HADS: Hospital Anxiety and Depression Scale; PCS: Pain Catastrophizing Scale; PSEQ: Pain Self-Efficacy Questionnaire; NPP: nuclear power plant; ESAS: Edmonton Symptom Assessment Scale; GHQ-12: General Health Questionnaire-12; CPSQI: Chinese Pittsburgh Sleep Quality Index; GDS: Geriatric Depression Scale; SOL: Sleep Onset Latency; WASO: Wakening After Sleep Onset; EMA: Early Morning Awakening; TST: Total Sleep Time; TWT: Total Wake Time; SE: Sleep Efficiency; C1: subjective sleep quality; C2: sleep latency; C5: sleep disturbances; C7: daytime dysfunction; CBT-I: Cognitive Behavioral Treatment for Insomnia; NAWK: number of awakening; SF-12: Short Form Health Survey-12 item; BDI-II: Beck Depression Inventory II; SRS: Stress Reactivity Scale; TBI: Traumatic Brain Injury; NSI: Neurobehavioral Symptom Inventory; C6: sleep medication of PSQI; PCL-M: modified post-traumatic stress disorder Checklist-Military Version; BSI-18: Brief Symptom Inventory; GAD-7: Generalized Anxiety Disorder Scale-7; SDQ: Sleep Disorder Questionnaire; PPV: positive predictive value; NPV: negative predictive value; PLMD: periodic limb movement disorder.

### 3.3. Excessive Daytime Sleepiness

Daytime sleepiness is defined as difficulty in maintaining the alert awake state during the wake phase of the 24-h sleep-wake cycle. Daytime sleepiness is an important manifestation of sleep disorders and it impacts the patient’s social life and threatens public health and safety [5]. Excessive daytime sleepiness (EDS) can be caused by disorders such as Obstructive Sleep Apnea (OSA), narcolepsy, and idiopathic hypersomnia [5,8]. Although MSLT and the maintenance of wakefulness test (MWT) [1] exist as objective measurements of daytime sleepiness, in sleep research and the clinical setting the Epworth Sleepiness Scale (ESS) is the most commonly used measure of sleepiness [74]. In the development of the ESS, the sleep disorders experienced by participants were primary snoring, OSA syndrome, narcolepsy, idiopathic hypersomnia, insomnia, and periodic limb movement disorder [74]. Thus, the ESS appears to be a convenient, standardized, and cost-effective way to measure sleepiness in patients who suffer from sleep disorders.

The ESS requires people to rate their likeliness of falling asleep in eight different situations (i.e., reading, watching TV, sitting in public, being a car passenger, resting in the afternoon, talking to someone, sitting quietly after lunch, stopping in traffic), chosen to represent different levels of somnificity that most people encounter as part of their daily lives [74]. Subjects base their ratings on the recent past, using a 4-point Likert scale (0: would never doze; 3: high chance of dozing). The term somnificity refers a general characteristic of a posture, activity, and situation with a capacity to facilitate sleep-onset in the majority of participants [74,75]. Each ESS item is scored as a number from 0 to 3 according to the individual’s response, and these scores are summed to determine a total ESS score, which ranges between 0 and 24. The higher the score, the higher the person’s level of daytime sleepiness. A cut-off value of 10 (ESS total score > 10) is usually considered to detect EDS [75]. In selected papers, the major concerns regard item 8. For example, in the Japanese version of the ESS there was some misunderstanding as to whether the question referred to being in a car as the driver or as a passenger [76]. Thus, the authors tested different alternatives and replaced item 8 with the following item: “while sitting and writing by hand”. The same procedure was adopted for item 1 (“while sitting and reading a book”) because this item remained largely without response. Thus, it was replaced by the following item: “reading something while sitting in a chair (newspapers, magazines, books, documents, etc). In a similar way, Rosales-Mayor et al. [77] proposed two versions: one for drivers and another for non-drivers. In the last case, item 8 was replaced by the item “standing and leaning or not on a wall or furniture”. By contrast, the 4-point Likert scale was adopted by all the papers, and the adequacy of this scale was also confirmed with the Rasch model [78,79].

In general, a 1-factor model (mean factor loading range 0.54–0.73) was confirmed [76,78,79,80]. The unidimensionality was also found in patients with sleep-disordered breathing [81] with factor loadings greater than 0.60, and was confirmed using Rasch analysis (explained variance of 39.92% and factor loading greater than 0.50) [82]. However, this factor structure was challenged by four articles supporting the 2-factor solution, with a mean explained variance equal to 56.06% [77,81,83,84], even if these two factors were loaded by different items (e.g., F1 with items from 1 to 4; F2 with items 5 to 8). Observing Table 3, the factor structure of the ESS seems to depend on the sample involved in the study, with different organization of items within both factors [77,81,83,84]. For example, [82] found that items 1, 2, 3, 4, 5, and 7 were grouped into Factor 1 and items 1, 3, 4, 6, and 8 were grouped into Factor 2, suggesting that several items loaded in both factors. Finally, in a very large sample, Lapin et al. [85] proposed a different factor solution with an improvement of the variance explained from the 1-factor (63.4%) to 3-factor (75.4%) models, with an improvement of the factor loadings from 0.505 to 0.995. In the 3-factor solution, Factor 1 was composed of items 3, 4, 6, and 8, Factor 2 comprised items 2 and 3, and item 5 made up Factor 3. As reported before, the factor structure of the ESS was related to the numerosity of the sample, the type of the sample, and the statistical method used, putting into question the reliability of the total score for detecting EDS [75].

As regards reliability, the ESS demonstrated a mean Cronbach’s alpha of 0.82, and the mean corrected item-total correlations ranged from 0.38 to 0.69, considering both original and modified ESS versions [76,77,78,79,80,81,82,83,84,85]. Even if in the majority of studies the critical item was item 8, only in one study was an increase of Cronbach’s alpha reported, with the exclusion of item 7 [83]. The internal consistency of the questionnaire was further found when analyzing test-retest reliability (mean ICC = 0.84), with a temporal interval from 7 to 35 days. Wu et al. [80] reported a split-half reliability coefficient in line with the reported ICC. The ESS appeared to be moderately associated with different PSG variables, such as AHI (Apnoea-Hypopnoea Index), lowest SaO_2_ (Oxygen Saturation), and mean SaO_2_ [78,81]. Single items correlated with PSG parameters, as reported in Table 3 [84]. Importantly, the ESS negatively correlated with another sleep questionnaire, the Functional Outcomes of Sleep Questionnaire (FOSQ; from −0.22 to −0.92), and with a healthy related quality of life (from −0.12 to −0.24), but not with the Life Orientation Test [78,80,83]. Finally, the total ESS score was associated with Body Mass Index (BMI) [78,80], even if this was not systematically confirmed [81].

In general, the articles reviewed reported known-group differences on the basis of AHI criteria (with an increase in total score as AHI increased) [78,84] and PSQI daytime dysfunction score (increase in ESS score as PSQI score increased) [76]. The OSA patients reported a higher total ESS score compared to healthy controls [79,81,82]; insomnia patients also reported a higher EDS than normal individuals [79]. Individuals with a medium level of education and with a normal BMI value reported a lower total ESS score than individuals with a lower or higher level of education and with a higher BMI value [80]. Even if, in our selected articles, no studies performed a ROC curve analysis to test the sensibility and specificity of the proposed cut-off for detecting EDS or proposed clinical cut-off values, four articles reported a clear responsiveness given that patients with OSAS who underwent treatment with Continuous Positive Airway Pressure (CPAP) for different durations (from 1 month to 6–9 months) improved in their ESS score with a relevant drop in the total score after treatment compared to before [76,77,81,84]. The changes in the total ESS score were greater in patients with severe OSAS.

## 4. Discussion

This comprehensive literature search identified 49 studies evaluating psychometric properties and latent factor structure of different questionnaires that measure sleep quality in adult populations. The studies were selected only when it was possible to extract the dimensionality (e.g., EFA, CFA, PCA, or Rasch model), the reliability (Cronbach’s alpha and/or test-retest coefficients), construct validity (convergent/divergent correlations and/or known-group differences), and eventually other information such as ROC curve analysis or responsiveness to clinical treatment. In the present paper, we review studies from 2008 to 2020 featuring the sleep questionnaires reported by Martoni and Biagi [22], including only those questionnaires (PSQI, AIS, ISI, MSQ, JSS, LSEQ, SLEEP-50, and ESS) with the aforementioned criteria. After observing the reliability and the validity of the selected sleep questionnaires, we can confirm that sleep quality is a complex construct which covers both sleep difficulties and daytime impairment. Although all selected tools, with the exception of the ESS, contain items assessing night-time sleep to a different extent (e.g., SLEEP-50), the MSQ [66] is the only self-report scale with a clear distinction between nighttime sleep disorder and daytime functioning/problems. Indeed, the remaining questionnaires have shown single components or factors assessing daytime impairment due to poor sleep quality (e.g., C7 for PSQI [21,25,26,30,31,32,33,34,36,38,39], daytime dysfunction factor for AIS [43,48,49,50], Impact of Insomnia factor for ISI [56,58,59,60,61,62,63], Behavior following awakening factor for LSEQ [70], or impact of sleep complaints on daily functioning for SLEEP-50 [72,73]) or single items assessing the subjective feelings of fatigue and sleepiness despite receiving a typical night’s rest for JSS-4 [68]. By way of contrast, the ESS is intended to differentiate individuals with EDS from alert people and, thus, it measures sleep propensity in eight different daily situations or different levels of somnificity [74,75]. Even if Martoni and Biagi [22] also reported two other subjective measures of sleepiness, such as the Stanford Sleepiness Scale (SSS) [86] and the Karolinska Sleepiness Scale (KSS) [87], not only is the ESS one of the most used tools for the EDS [88] (as also demonstrated by the lack of articles from 2008 to 2020 with our inclusion criteria for SSS and KSS), but it is also different from the SSS and KSS. Indeed, the SSS and KSS are usually used to assess “state” sleepiness, that is, these scales are used to measure short-term changes in sleepiness [89] and are considered a measure of subjective feeling of drowsiness (or fatigue) [88]. Indeed, the SSS is based on a Likert-type scale with seven vigilance levels and people are asked to indicate which level best describes their current state. The KSS is based on a 9-point scale measuring the subjective level of sleepiness at a particular time during the day, in which individuals indicate which level best reflects the situational sleepiness. In contrast, the ESS measures a global level (daily life soporific situations) of sleepiness, that is a “trait” aspect of sleepiness [74,75,89]. Thus, sleep quality should be evaluated using a combination of the different tools reviewed, in order to obtain a complete picture of both sleep and daytime impairments.

Taking into account the importance of dimensionality, reliability, and validity for research, epidemiological, and clinical studies, we discuss the results below under the domains of factor structure, reliability, and construct validity. In general, a clear and unique factor structure was not defined for any of the self-reported questionnaires included, with the exceptions of MSQ [66,67], JSS [69], and SLEEP-50 [73]. As reported in a previous review [90], the structured categorical data of the PSQI could be sensitive to the specific model (method of extraction) being applied. The lack of a defined factor structure for the PSQI could be related to the fact that parsimony is not applied and EFA or CFA are not used (with a concomitant lack of reporting of relevant details). In addition, the heterogeneity of sample analyzed (with highly variable characteristics in terms of societal stressors, medical pathology, sleep medication use, pain, etc.), the small size of the sample, the proposed modified versions, and the cultural translation limited any interpretation of the different factor structure, and called into question the value of the global PSQI score in detecting poor and good sleepers. In a similar way, the factor analyses performed on the ISI scale demonstrated a multidimensionality of the questionnaire. In this case, it is worth noting that the 3-factor solution was found in European (Spanish, Italian, German, and Danish) individuals, probably reflecting a more typical characteristic of the clinical disorder in these cultures [56,61,62,63]; the 2-factor solution was more widespread in different cultures from Africa [60] to Asia [58,59], through Europe [61]. However, unidimensionality was found in a large sample (in total 2887 participants), including both patients (cancer, chronic pain, and traumatic brain injury; [47,54,57]) and community-dwelling individuals such as students and employees [55,56], putting the real dimensionality of the self-report questionnaire into question. In the 2-factor solution it was constantly confirmed that items 6 and 8 seemed to indicate a daytime dysfunction, reflecting the association between sleep and wake (i.e., a poor night’s sleep impacts on subsequent daytime functioning and a stressful daytime impacts on the subsequent night’s sleep). In a similar way, the ESS mainly showed different factor solutions. Moreover, many concerns regarded item 8 (“in traffic”), probably due to a general misunderstanding of whether it means being in the car as a driver or a passenger [77]. Finally, the single reviewed study concerning the LSEQ did not confirm any factor solution because several solutions showed a best fit with some indices but not with others, bringing into question the real latent structure underlying the LSEQ [71]. Altogether, these results shed light on the necessity of the procedural details (e.g., EFA and CFA in the same article) and application of standard practices in order to streamline the debate on the heterogeneity of self-report questionnaires, with the main goal being to disentangle the meaning of a total (or more than one) (sub-)score for clinicians. In contrast, the MSQ, JSS, and SLEEP-50 showed a clear factor structure. These findings seemed to suggest the applicability of these various factor structures with a clinical application, although the SLEEP-50 seemed to be more time-consuming for both respondents and the scorer. However, studies investigating the factor structure of the MSQ, JSS, and SLEEP-50 in the past 12 years have been scarce and thus we recommend further studies aimed at confirming the unidimensionality of the JSS, the 2-factor solution of the MSQ. and the multidimensionality of the SLEEP-50.

As regards the psychometric properties, the present review showed that all self-report questionnaires reported good reliability, with a Cronbach’s alpha greater than 0.70 (Table 1, Table 2 and Table 3) [18,19], even if in eight articles (six for PSQI, one for AIS, and one for MSQ) the Cronbach’s alpha was <0.70. In general, the reviewed findings indicated that the included questionnaires showed a positive rating for within- and between-group comparisons. Importantly, five studies [57,61,69,73,85] reported Cronbach’s alpha within the ideal range for comparison of individual scores (i.e., 0.90–0.95). These results could support the notion of sleep quality being based on both reflective and formative models [91]. The consistency of the selected scales was also confirmed by analysis of the internal homogeneity of the questionnaire. Indeed, all self-reported questionnaires demonstrated a corrected item-total correlation in the range of 0.30–0.86. In addition, the test-retest showed good reliability of all tools in different temporal intervals (from 1 week to 6/9 months), supporting the stability of the dimension being measured between the test and the retest. However, we observed that 19 out of 49 selected studies (roughly less than 40%) performed a test-retest correlation and thus we recommend further research to establish the test-retest of the self-reported questionnaires (especially for JSS, LSEQ, and SLEEP-50) over different time periods, considering that the time periods (past month, past 2 weeks, past week, or habitual experience) covered by selected tools differ.

The basic principle of construct validation is that hypotheses are formulated about the relationship between scores of the instrument of interest and scores of other instruments measuring a similar or different construct. The published findings we assessed outlined high correlations of the PSQI with other measures of sleep quality (e.g., ISI) or sleep measures [29,31,38], while it reported weak or no associations with other measures such as ESS [29,31]. As far as convergent validity is concerned, the PSQI showed moderate associations with depression and general quality of life, supporting the similarity in constructs between scales, and reinforcing evidence from clinical epidemiological studies that document high degrees of comorbidity between sleep and psychiatric disorders [92]. It is possible that altered mood states (e.g., depression and anxiety) may influence the perception of one’s physiological state, including somatic symptoms [13,93], and, hence, the associations observed which were based on participants’ self-report may be subject to bias. However, the poor correlations among objective measures of sleep quality (e.g., PSG or actigraphy) and the PSQI (Table 1; [32,36]) confirm previous studies [21]. This reduced level of concurrent validity could be explained by different aspects. First, daily fluctuations of sleep cannot be described by a questionnaire investigating sleep quality over the past month. Second, the dissociation between objective and subjective measures of sleep could be due to different aspects such as sleep setting, personality traits, and constitutional factors [94]. Third, the self-report questionnaire is very vulnerable to memory processes (especially remembering information from the past 4 weeks) and misperception, with the tendency to exaggerate number and gravity of symptoms [1]. Among the sleep disorder scales (Table 2), the AIS in its two versions showed convergent validity and had moderate associations with different sleep scales (PSQI and ISI) and with anxiety and/or depression [45,46,47,48,49,50]. On one hand, these results confirmed that the selected self-report tools converged towards a similar construct of subjective sleep quality. On the other hand, the strong comorbidity between sleep and mood alterations, and sleep and psychological well-being is further confirmed [13,93,94]. A similar pattern, with moderate-to-strong correlations, was also found for the ISI, reflecting a true sleep disturbance construct. However, the ISI did not correlate with ESS [59,62], even if daytime sleepiness can be caused by overnight insomnia, and studies had shown that not only do insomniacs not sleep effectively at night but also have difficulty falling asleep in the daytime [95]. Moreover, the ISI score and the three specific items reported small correlations with PSG and sleep diary variables such as WASO, EMA (Early Morning Awakening), TST, TWT, and SE [59,63]. As before, the subjective and objective measurements of sleep do not show proper correlations, probably due to the discrepancy between the recall period of the ISI and the single night of a PSG recording [96]. In addition, the crucial role of the first three items could indicate how these items, which investigate three different subtypes of insomnia, are useful for the diagnosis and the assessment of insomnia, while the remaining items (5–7) are independent factors assessing daytime effects of insomnia. The remaining sleep disorder scales showed low-to-moderate correlations with PSQI and perceived stress or psychological distress [67,69,73], but the small number of studies that have used the MSQ, JSS, or SLEEP-50 in the past decade casts doubt as to their construct validity. Finally, the ESS showed a weak-to-moderate association [78,81,84] with different PSG variables (AHI, lowest SaO_2_, mean SaO_2_, mean latency to sleep, mean latency to REM sleep, and number of times the patient falls asleep), in line with what has been reported in a previous review [88]. These findings may reflect that a comprehensive evaluation of sleepiness may require multiple measures, high heterogeneity of studies included, and diversity within target populations [88]. The negative correlations between ESS and FOSQ or Short Form Health Survey-36 (SF-36) dimensions indicated that higher daytime sleepiness was related with functional impairments in a broad range of activities, with a main impairment in activity level, vigilance, and social outcome. Daytime sleepiness seemed to influence more or less all parts of life to such an extent that people with the condition perceive themselves as being generally more limited by their health than those without it [78,80,83]. However, it remains to state that few studies tested a priori hypotheses related to the relationship between sleep quality, insomnia or daytime sleepiness, and other constructs by stating the expected direction and magnitude of associations beforehand, based on what was known about the construct under study. Thus, we recommend an assessment of similarity and/or dissimilarity between constructs with formulated hypotheses set a priori. This formulation could be created with the content of utilized tools and a clear description of what is known about the population in the study [11].

Further evidence of construct validity can be observed in the results derived from known-group differences, with strong value for clinical practice. As shown in Table 1, the PSQI demonstrated robust known-group validity based on both proposed cut-off points and other sleep disorder assessments. In addition, the discrimination between good and poor sleepers was found according to different cut-off scores of psychological or medical variables. These results were further confirmed by regression analyses which revealed that depression, anxiety, and stress predicted poor sleep quality [34,38]. However, few studies performed ROC curve analysis, and future investigation should test the critical points for distinguishing poor and good sleepers, especially when a multidimensional factor structure is proposed. Table 2 also confirmed known-group validity of AIS in different target populations [45,46,47,48,49,50], of ISI for different criteria [47,58,59,61], of MSQ subscales [66] in detecting hypersomnia and insomnia problems (or compared to PSQI [67]), of JSS-4 in proposing normative values, and of LSEQ [71] and SLEEP-50 [73] as standardized tools for screening multiple sleep complaints. Importantly, for both AIS and ISI the proposed cut-off values allowed for the discrimination between insomniacs and non-insomniacs with an objective confirmation using actigraphic data [47]. However, only 9 out of the 21 studies included performed the ROC curve analysis, thus not only limiting the possibility of testing the sensibility and specificity of original cut-off points in different cultures and population but also of comparing different tools with each other in terms of validity. Future studies are needed to bridge this gap. In a similar way, the ESS demonstrated a strong ability to detect individuals with differences in daytime sleepiness, such as OSA and narcolepsy patients [76,77,78,80,81,82,84,85]. In addition, we found four articles which clearly demonstrate a responsiveness of the ESS to CPAP treatment with a significant drop in the total score, suggesting that the ESS is able to discern the severity of OSAS [76,77,81,84]. However, in our review we did not find any study that performed a ROC curve analysis in order to test the cut-off points for the detection of the EDS: these types of studies are recommended.

The present review has potential limitations. For example, the psychometric properties were not appraised using the “Consensus-Based Standards for the Selection of Health Status Measurement Instruments” (COSMIN) checklist, an instrument developed to evaluate the methodological quality of studies on measurement properties of health-status related questionnaires [97], even if the COSMIN approach may set standards that are too high to achieve a good rating on some of the criteria [98]. Another limitation could be related to the choice not to perform a meta-analysis, but the discrepancies made this almost impractical. Although we recommend that these limitations are taken into account in the future, we obtained and presented data in terms of factor structure, reliability, and construct validity in a similar way to the reviews from which the COSMIN method was adopted [7,8,9,10,11,12,88,89,90]. In addition, this present qualitative review allows for a picture of the psychometric properties of different tools to be obtained, taking into account the heterogeneity of the reviewed methods, populations, and statistical analyses, which could be considered a potential limitation for the meta-analysis. Finally, we have updated the review published in 2007 by Martoni and Biagi [22], in which 26 self-report questionnaires were reviewed. In this way, we have reported the factorial structures and psychometric properties of sleep quality questionnaires that have emerged during the past 12 years, suggesting their usefulness in epidemiological, research, and clinical settings.

## 5. Conclusions

In summary, sleep quality is a multifactorial construct that is difficult to define and measure objectively, considering the great variability between individuals and its subjective nature. In the present review, we focus on those which have been validated in terms of internal consistency, construct validity, and latent factor structure. The PSQI, the most widely used questionnaire for subjective sleep quality [21], demonstrated good reliability and validity, especially for known-group validity. However, questions regarding its factor model, the large recall period, and the scoring system challenge the value of the global PSQI score for distinguishing poor and good sleepers. Several sleep disorder questionnaires have been proposed; complaints about sleep quality are common and poor sleep quality can be an important symptom of many sleep disorders. These tools reveal good psychometric properties and easier administration and scoring (but not for the LSEQ and SLEEP-50). Additional studies are needed, on one hand, in order to clarify the factor structure of AIS and ISI and, on the other hand, to add to the evidence regarding the validation of both MSQ and JSS in epidemiological, research, and clinical use, considering that both are inexpensive, and easy to administer, complete, and score. Finally, the ESS showed good internal consistency and known-group construct validity was established. Questions remain concerning the factor structure of the ESS scale and about the definition of cut-off scores for clinical use. In conclusion, all self-report questionnaires assessing subjective sleep quality require further studies of high methodological quality to assess their measurement properties, not only in terms of reliability and construct validity but also in terms of latent structure and discriminability. In particular, studies are required in the area of test-retest reliability as well as responsiveness to change. Within the subjective questionnaires included, the MSQ appears to be most suitable with which to ascertain the presence of sleep disorders (i.e., insomnia disorder) and the negative daytime consequences due to poor sleep (e.g., daytime sleepiness) with a complete assessment of the sleep-wake cycle. In addition, it is inexpensive and easy to administer, complete, and score, with good psychometric properties. A recently developed tool, the Sleep Condition Indicator (SCI) [99] with the diagnostic criteria of the Diagnostic and Statistical Manual of Mental Disorders, Fifth Edition (DSM-5) [100] could also be considered in future studies. Indeed, the SCI is a short tool (8-item), and is easy to administer (each item is scored on a five-point scale) and easy to score (total score from 0 to 32, with higher values indicating better sleep). The SCI is reliable (Cronbach’s α = 0.73, mean item-total correlation ranges from 0.42 to 0.50, ICC = 0.84) and valid (gender differences) with a specific cut-off of 7. The assessment of its dimensionality and a further investigation of its construct validity are recommended.

## Figures and Tables

**Table 1:** Reported measurement properties of PSQI: dimensionality, reliability, construct validity, and Receiver Operating Characteristic (ROC) curve analysis.

			Reliability		Construct Validity			
Study and Abbreviation of Questionnaire	Population	Dimensionality	Internal Consistency	Test-Retest	Convergent/Divergent Validity	Known-Group Validity (Mean Value)	Cut-Off Score or ROC Curve	Version
Chien et al., 2008 [25]: SC_PSQI	A total of 3742 workers (2091 females); mean age of 27.66 years	Infit and Outfit mean squares between 0.60 and 1.40 indicating a single construct for sleep quality	Cronbach’s alpha of 0.80 (person separation reliability of 0.75)		There was an agreement between SC_PSQI and PSQI classifications (Cohen’s Kappa = 0.864	Insomnia group (cut-off > 11; about 12) > SubCriteria group (cut-off > 5; about 9) > no disease group (score < 5; about 1)	PSQI > 5 (severe) and PSQI > 11 (very severe)	Chinese
Skouteris et al., 2009 [30]: PSQI	A total of 252 pregnant women; mean age of 31.67 years	2-factor correlated model with a path from overall sleep quality to both of the factors: F1 or Sleep Efficiency: C2, C3, C4; F2 or Night and Daytime Disturbance: C5, C7.Inter-factor correlations were 0.44 and 0.59 at both Ts:	Cronbach’s alpha at T1 (between 15–23 weeks gestation time) of 0.70. Cronbach’s alpha at T2 (between 29–39 weeks gestation time of 0.76. Removing C6 Cronbach’s alpha improved to 0.72 and 0.78 for T1 and T2, respectively. Item-total: correlations ranged from 0.30 to 0.72	For PSQI total score r = 0.56, with Sleep Efficiency r = 0.45, Night and Daytime Disturbances r = 0.52 and Sleep quality r = 0.39	PSQI did not correlate with gestation at either time points (*p* > 0.05). PSQI score did not correlate with age (all r < −0.05) or education (all r < −0.11). At T1, PSQI total score (r = 0.47), F1 (0.29), F2 (0.45) and Overall sleep quality (0.47 correlated with BDI. At T2 PSQI score (0.36), F1 (0.26), F2 (0.38) and overall sleep quality (0.46) correlated with BDI	No depressive symptoms (5.37) < mild depressive symptoms (7.75) < moderate/severe depressive symptoms (9.13) for PSQI score, sleep efficiency (respectively, 1.72, 2.47, 3.31), night and daytime disturbances (respectively, 2.56, 3.53, 3.69) and overall sleep quality (respectively, 2.09, 2.57, 2.94). At T1 and T2 sleep quality was the only significant predictor of BDI scores. Sleep efficiency and night and daytime disturbance (without enthusiasm item) at T1 predicted BDI scores at T2		English (Australian)
Kotronoulas et al., 2011 [31]: GR-PSQI	A total of 209 patients with a diagnosis of cancer (124 females); mean age of 54.85 years	A 2-factor model (59.20% of the variance explained):F1 or Quality of Nocturnal Sleep: C1, C2, C3, C4, C5; F2 or Daily Disturbances and Management of Sleep Problems: C6, C7	Cronbach’s alpha of 0.76. Reliability increased to 0.78 omitting C6. Cronbach’s alpha of F1 was 0.80. Cronbach’s alpha of F2 was 0.40 Component-to-global score correlations ranged from 0.43 to 0.78. Component-to-total factor score ranged from 0.54 to 0.85 for F1 and from 0.75 to 0.79 for F2	14–21 days: Global GR-PSQI r = 0.82; Factor1 score r = 0.81; Factor2 score r = 0.64	GR-PSQI × SQ-VAS (r = −0.75); GR-PSQI × ISI (r = 0.81);GR-PSQI × ESS (n.a. but *p* > 0.05). F1 × SQ-VAS (r = −0.73); F1 × ISI (r = 0.80) F1 × ESS (n.a. but *p* > 0.05.). F2 × ESS (0.30) F2 poorly correlated with SQ-VAS and ISI	Low anxiety (7.03) > high anxiety (11.29) for GR-PSQI, F1 (5.61 and 8.45) and F2 (1.42 and 2.84) scores. Low depression (7.60) > high depression (11.17) for GR-PSQI, F1 (6.08 and 8.21) and F2 (1.51 and 2.96) scores. Patients with poorer performance status (14.80) > patients with high performance status (7.93) for GR-PSQI, F1 (10.80 and 6.31) and F2 (4.00 and 1.62) scores	PSQI > 5	Greek
Sohn et al., 2012 [32]: PSQI-K	A total of 133 healthy controls (99 females; mean age of 40.02 years), 211 insomnia (134 females; mean age of 52.50 years) patients and 50 narcolepsy (22 females; mean age of 26.72 years) patients	A 2-factor solution: F1: C1, C2, C3, C4, C6 (loading range 0.693–0.860). F2: C5, C7 (loading range 0.634–0.880)	Cronbach’s alpha of PSQI-K was 0.84. Component-to-total score ranged from 0.40 to 0.83 (in healthy controls 0.32–0.62 range; in insomnia 0.16–0.72 range; in narcolepsy 0.21–0.68 range)	At 2–4 weeks later test-retest correlation coefficient was 0.65 for total score and for the seven components from 0.30 to 0.84.No significant difference was found between the two values using the paired t-tests. In the retest, Pearson’s correlation between the total score of the PSQI-K and the components of the PSQI-K showed correlation scores from 0.42 to 0.69 with a mean of 0.58	There was a weak correlation between sleep latency obtained through PSG and sleep latency assessed using the PSQI-K (r = 0.225). The same occurred between sleep efficiency obtained with PSG and habitual sleep efficiency assessed using the PSQI-K (r = −0.331). The other sleep variables of the OSG had no significant correlation with the PSQI-K	Insomniacs (14.71) > narcoleptics (8.40) > healthy controls (4.06). Subjects with insomnia had higher scores for all components except for daytime dysfunction than those with narcolepsy or healthy controls. Patients with narcolepsy had higher scores than controls in four components except for sleep latency, sleep duration and use of sleeping medication	PSQI > 8.5; ROC curve with sensitivity of 0.943 and specificity of 0.844	Korean
Curcio et al., 2013 [36]: PSQI	A total of 10 healthy controls (five females; mean age of 26.0 years), 10 healthy elderly people (five females; mean age of 68.6 years), 10 patients with dementia (five females; mean age of 75.0 years), 10 patients with OSA (two females; mean age of 67.1 years), 10 patients with depression (five females; mean age of 53.0 years)	3-factor model: Perceived Sleep Quality: C1, C2, C6; Sleep Efficiency: C3, C4; Daily Disturbances: C5, C7	Cronbach’s alpha of 0.835		There were correlations between Stage 2 latency and PSQI (r = 0.294), between SWS latency and PSQI (r = 0.524), between Stage 1% and PSQI (r = 0.327) and between Stage 2% and PSQI (r = −0.349). No other PSG sleep variables correlated with PSQI	Healthy controls (4.00) or healthy elderly (3.90) < patients with dementia (7.60) < OSA (11.20) or patients with depression (12.60). The same pattern for all components, except C7, and all three factors	PSQI > 5; ROC curve with AUC = 0.705, sensitivity = 0.939, specificity = 0.471, +LR = 1.77 and –LR = 0.13	Italian
Panayides et al., 2013 [23]: PSQI-G	A total of 600 Cypriots (319 females); age range 18–65+. 20 patients with depression (eight females); age range 23–75	Original PSQI-G: dimensionality (35.5% of the variance) and the ratio of variance explained by the measures to the variance explained by the first component was 4.65:1; infit and/or outfit values greater than 1.4. Modified 16-item scale of PSQI-G: dimensionality (47.2%) and the ratio of variance explained by the measures to the variance explained by the first component is 6.2:1; infit and outfit values < 1.4	Original PSQI-G: the person reliability was low at 0.69 as was the person separation at 1.48. Modified 16-item PSQI-G: the person reliability was 0.84, the person separation 2.25 and item reliability was 0.99. Items exhibited satisfactory point measure correlations between 0.44 and 0.67			Depression patients had higher scores (21.35) and measures (0.2915) than non-clinical sample (15.73 and −0.5840, respectively)	Probability curves for the 3 categories: category 0 was the most probable for measures lower than −0.68; category 1 for measures between −0.68 and 0.68 (range of 1.36 logits); category 2 was the most probable for persons with measures above 0.68	Greek
Hita-Contreras et al., 2014 [33]: PSQI	A total of 138 women with fibromyalgia; mean age of 52.87 years	A 2-factor solution (54.96% of the variance explained): F1: C1, C2, C3, C4, C7 (loading range 0.449–0.842); F2: C5, C6 (loading range 0.798–0.811)	Cronbach’s alpha of 0.805 for PSQI, 0.866 for F1 and 0.712 for F2. Corrected item-to-total correlation ranged from 0.380 and 0.667	2-weeks test-retest: Significant correlations for PSQI total score (Spearman coefficient = 0.806, for F1 (0.687) and for F2 (0.659). All components correlated with each other (range 0.356–0.718)	Correlations were found between PSQI and FIQ (0.304), SF−36 physical functioning (−0.372), SF-36 role physical (−0.217), SF-36 and role emotional (−0.254), SF-36 vitality (−0.247), SF-36 mental health (−0.208), SF-36 social functioning (−0.426), SF-36 bodily pain (−0.351), PHS (−0.403) and MHS (−0.392), but not General Health. Daytime dysfunction, mirrored the same correlation pattern while other components were correlated with scales in a sporadic way	Moderate severity of fibromyalgia (12.05) < severe fibromyalgia (14.39 for the total score and all components, except C2, C5, and C6)		Spanish
Zhong et al., 2015 [38]: PSQI	A total of 642 women at ≤16 weeks of gestation; mean age of 28.8 years	A 3-factor model (60.10% of variance explained): F1 (C5, C2, C7, C1), F2 (C3, C4), F3 (C2, C1, C6) with correlation between F1 and F2 and between F1 and F3. 2-factor model excluding C6 and with C1 loading on 1 factor demonstrated a good fit	Cronbach’s alpha of 0.57 for global PSQI score. Item-total correlations ranged from 0.10 to 0.40		Adjusted (for maternal age) correlations were present between global PSQI score (with and without C6), F1, F2 and F3 with FIRST (0.35–0.47 excluding F2), PHQ-9 (0.11–0.52) and GAD-7 (0.11–0.49)	Multivariable logistic regression analyses found that poor sleep quality was associated with 3.57-fold increased odds of susceptibility for stress-induced sleep disturbance and that poor sleepers had 5.48-fold increased odds of depression and 4.57-fold increased odds of generalized anxiety disorder	PSQI ≥ 5	Spanish (Peruvian)
Anandakumar et al., 2016 [26]: PSQI	A total of 205 patients attending the outpatients department of a tertiary care hospital (140 females); mean age of 50.2 years	A 1-factor solution (53.53% of variance explained) with loading factor from 0.50 to 0.83	Cronbach’s alpha of 0.85. Corrected item-total correlations ranged from 0.42 to 0.81			Depression (CES-D ≥ 16) patients reported higher PSQI score (10.13) than non-depressed patients (4.47). The same pattern was found for all components		Sinhala
Qiu et al., 2016 [34]: PSQI	A total of 1488 pregnant women enrolled in the Migraine and Pregnancy Study; mean age of 33.4 years	A 2-factor model (52.8% of variance explained): F1 (C1, C2, C3, C4) and F2 (C5, C7) with correlation between C3 and C4 (0.43) and between C2 and C4 (0.09)	Cronbach’s alpha of 0.74. Corrected item-total correlations ranged from 0.31 to 0.64		There were Spearman’s rank-order correlation coefficients for PSQI with and without C6 and PHQ-9 (0.478 and 0.468), DASS-21 total score (0.421 and 0.416), DASS-21 anxiety (0.294 and 0.292), DASS-21 depression (0.378 and 0.373). and DASS-21 stress (0.367 and 0.362)	Multivariable logistic regression analyses found that poor sleepers had increased odds of experiencing depression (OR = 6.47) assessed using PHQ-9, had 4.34-fold increased odds of depression (DASS-21 depression), 3.59-fold increased odds of anxiety and 4.37-fold increased odds of stress	PSQI score > 5	English (American)
Becker and de Neves Jesus, 2017 [39]: PSQI-PT	A total of 204 community-dwelling older adults (152 females); mean age of 70.05 years	A 2-factor model (40.575% of variance explained): F1 (C1, C2, C3, C4) and F2 (C5, C7) with inter-factor correlation of 0.360. 3-factor model with better fit: Perceived Sleep Quality (C1, C2), Sleep Efficiency (C3, C4) and Daily Disturbances (C5, C7). Correlations between Perceived Sleep Quality and Sleep Efficiency (0.63), between Perceived Sleep Quality and Daily Disturbances (0.56) and between Sleep Efficiency and Daily Disturbances (0.46)	Cronbach’s alpha for global sleep quality was 0.69 but increased to 0.70 when C6 was excluded. Inter-component correlations ranged from 0.12 and 0.52. Component-total correlations ranged from 0.32 to 0.55 except for C6 which was 0.24			Regression analysis showed that gender (male = 4.76 vs. females = 6.39) and self-assessed healthiness (those who said they considered themselves healthy = 5.23 vs. those who did not = 9.42) had a higher global PSQI score	PSQI > 5	Portuguese (European)
Del Rio João et al., 2017 [27]: PSQI-PT	Convenience sample of 347 community-dwelling adults (114 female); mean age of 35.93 years	A 1-factor solution (26.472% of variance explained) with factor loadings from 0.376 to 0.608	Cronbach’s alpha of 0.70. Inter-component correlations ranged from −0.001 to 0.41. Component-total correlations ranged from 0.46 to 0.61			Regression analysis found that age (> 46 years = 5.35 vs. 18–27 age = 6.78), and literacy (master’s and PhD degrees = 5.24 vs. basic scholarship completed = 6.27) were predictors of the global score		Portuguese (European)
Salahuddin et al., 2017 [40]: PSQI	A total of 311 adults from Mizan-Aman town, Southwest Ethiopia (35 females); mean age of 25.45 years	A 1-factor model > 40% cumulative variance) excluding C6, C7. A 2-factor model > 40% cumulative variance) with 7 (F1: C1, C2, C3, C4, C5; F2: C6, C7) or 5 (F1: C1, C2, C5; F2: C3, C4) components. A 3-factor model with F1 (C1, C2, C5), F2 (C3, C4) and F3 (C6, C7). All models performed poorly for different indices of confirmatory factor analysis, indicating no better fit for one single model	Cronbach’s alpha of 0.59 which increased to 0.62 when removing C6. Component-total correlations ranged from 0.15 to 0.81			Groups identified as normal sleep and insomnia using ICSD-R criteria across global PSQI score and all-components score except C6 and C7	PSQI > 5.5 ROC curve (AUC = 0.78) with sensitivity and specificity for all global PSQI scores between 0.5 and 16. The sensitivity = 82% and specificity = 56.2% of the PSQI cut-off	Ethiopian
Yunus et al., 2017 [28]: PSQI	Phase 1: 183 community-dwelling older Malaysians aged 60 or more (no gender information); Phase II: 1648 community-dwelling older Malaysians (992 females) with 60 or more years	A 1-factor model with only C1, C2, and C5 and factor loadings > 0.49	Cronbach’s alpha of 0.60. Item-total correlations ranged from 0.01 to 0.39	At 2-weeks test-retest: Original PSQI = 0.40 (ICC = 0.62), Malay version = 0.42 (ICC = 0.61). C1 = 0.17 (ICC = 0.29), C2 = 0.38 (ICC = 0.56), C3 = 0.38 (ICC = 0.55)		PSQI scores were higher in cases of neglect (4.11), followed by physical (4.10), psychological (3.96) and financial (3.60) abuse. No abuse (3.41) < 1 type of abuse (3.50) < 2 types or more (3.84). Generalized Linear Models showed that income (low and middle), self-rated health (poor), number of co-morbidities, gait speed, social support, and abuse were associated with higher PSQI scores		Malay
Gomes et al., 2018 [29]: PSQI-EP	A total of 564 adults (344 females) with 534 non sleep-clinic participants (324 females; mean age of 30 years) and 30 clinic sleep adults (20 females; mean age of 38.63 years)	A 1-factor solution (41.65% of variance explained) with factor loadings ranging from 0.525 to 0.775)	Cronbach’s alpha of 0.75. Corrected item-total correlations ranged from 0.37 to 0.62		Correlations were present between PSQI and ISI (0.797), PSQI and GSES (0.441) but not between PSQI and STOP-bang (0.162). PSQI components correlated with ISI (range 0.316–0.717); C1, C2, C5, C6, C7 correlated with GSES (0.425, 0.449, 0.356, 0.234, 0.324, respectively). No PSQI component correlated with STOP-bang (all r < 0.20)	Groups reporting the presence (8.76) versus the absence (4.72) of sleep problems for global PSQI score. Significant difference for all components except C4. The sleep-clinical group (12.67) had a higher PSQI score than the non-clinical group (5.12), and this difference was significant for each component	PSQI > 6 for self-reported; PSQI > 7 for clinical assessment with better balance between sensitivity and specificity relative to PSQI > 5 ROC curve for self-reported sleep problem with AUC = 0.87, sensibility = 85% and specificity = 70.1%. ROC curve on clinical sleep assessment AUC = 0.94, sensitivity = 86.7% and specificity = 84.5% with cut-off > 7 while with cut-off > 5 sensitivity = 100% and specificity = 64.6%c	Portuguese (European)
Morris et al., 2018 [41]: PSQI	A total of 198 participants (104 women) in the Diabetes Sleep Treatment Trials; mean age of females 55.3 years vs. males 58.5 years	A 1-factor structure with eigenvalues > 1 and 68.075% of cumulative variance for women and 74.111% of cumulative variance for men. 3-factor solution in women: F1 (C1, C2, C5, C7), F2 (C3, C4), F3 (C3, C6). 3-factor solution in men: F1 (C1, C2, C3, C4), F2 (C5, C7) and F3 (C2, C6) with factor loadings > 0.32	Cronbach’s alpha in total sample was 0.695 (men = 0.715; women = 0.674). Inter-item correlations showed none of the components to be above 0.80, indicating little or no redundancy. C4 correlated with C3 (0.608) in men, but less so in women (0.363). C3 was correlated to a greater extent with C1 in men (0.515) compared to women (0.245). C6 was the least correlated with any of the other components except for C2 and in many instances was not significant (values not reported)			Gender differences for C1 (male = 1.44 vs. female = 1.68) and C5 (male = 1.87 vs. female = 2.09)		English (American)
Seidi et al., 2019 [37]: PSQI-K	Pilot study: 50 undergraduate students (25 females; mean age of 20.21 years). Main study: 131 healthy participants (mean age of 20.11 years), 40 participants with insomnia complaints (11 females; mean age of 26.12 years) and 40 participants with physical symptoms (17 females; mean age of 25.32 years)	A 3-factor model with Sleep Quality (C2, C4), Sleep Disturbances (C3, C5) and Daytime Dysfunction (C1, C6, C7) with inter-correlations between factors: Sleep Quality × Daily Dysfunction = 0.70, Sleep Disturbances × Daily Dysfunction = 0.27, and Sleep Quality × Sleep Disturbances = 0.30	Pilot study: Cronbach’s alpha of 0.63. Main study: Cronbach’s alpha of 0.70; Cronbach’s alpha for each component: C1 = 0.98, C2 = 0.98, C3 = 0.75, C4 = 0.97, C5 = 0.66, C6 = 0.96, C7 = 0.82). Polychoric correlations among components ranged from 0.20 to 0.57	A 4–6 weeks test-retest with Spearman correlation coefficient of 0.83	Correlations appeared between PSQI-K and GHQ28 (0.72), as well as each PSQI-K component and GHQ28 (range 0.23–0.62)	Healthy participants (4.27) < insomnia (6.58) but not somatoform symptoms (4.95)		Kurdish
Al Maqbali et al., 2020 [35]: PSQI	A total of 369 patients with cancer (245 females; age range 18-more than 60)	A 2-factor model: Perceived Sleep Quality (C1, C2, C3, C6, C7) and Sleep Efficiency (C3, C4) with inter-factor correlation of 0.40	Cronbach’s alpha of 0.77. Component-total correlations ranged from 0.38 to 0.62			No gender differences in global PSQI score. Differences in global PSQI score for cancer site (higher scores for lung = 12.95, gastrointestinal = 12.05 and brain = 11.84), stage of diseases (IV stage = 10.69 > II stage = 9.45 > II stage = 8.61 > I stage = 8.32) and co-morbidities (yes = 10.36 > no = 8.18)		Arabic

Abbreviations: C1: perceived sleep quality, C2: sleep latency, C3: sleep duration, C4: habitual sleep efficiency, C5: sleep disturbance, C6: sleep medication use, C7: daytime dysfunction; BDI: Beck Depression Inventory; SQ-VAS: sleep quality visual analogue scale; ISI: insomnia severity index; PSG: polysomnography; SWS: slow wave sleep; ROC: receiver operating characteristics; AUC: area under the ROC curve; +LR: likelihood ratio for positive result; -LR: likelihood ratio for a negative results; FIQ: Fibromyalgia Impact Questionnaire; SF-36: Health Survey Short Form-36; PHS: Physical Health Summary; MHS: Mental Health Summary; FIRST: Ford Insomnia Response to Stress Test; PHQ-9: Patient Health Questionnaire-9; GAD-7: Generalized Anxiety Disorder-7; DASS-21: Depression, Anxiety Stress Scale-21; ICSD-R: International Classification of Sleep Disorders, revised criteria; GSES: Glasgow Sleep Effort Scale; GHQ28: General Health Questionnaire-28.

**Table 3:** Reported measurement properties of Epworth Sleepiness Scale (ESS): dimensionality, reliability, construct validity, and ROC curve analysis.

			Reliability		Construct Validity			
Study and Abbreviation of Questionnaire	Population	Dimensionality	Internal Consistency	Test-Retest	Convergent/Divergent Validity	Known-Group Validity (Mean Value)	Cut-Off Score or ROC Curve	Version
Izci et al., 2008 [78]: ESStr	A total of 150 subjects with SDB (38 females; mean age of 49 years) and 60 healthy controls (16 females; mean age of 43 years)	In individuals with sleep apnoea a 1-factor model was found (eigenvalue of 4.2 and 52% of variance explained). Factor loadings ranged from 0.43 to 0.76	Cronbach’s alpha was 0.87 for individuals with sleep apnoea and it was 0.86 for controls	A 4–5 week test-retest with correlation coefficient of 0.80 and ICC of 0.81. No difference in each item nor in the total score in the first and second assessment (8 vs. 8)	Correlations were found between AHI and total ESStr scores (0.44), between lowest SaO_2_ and ESStr (−0.45), and between mean SaO_2_ and ESStr (−0.30). In individuals with sleep apnoea there was no relationship between the ESStr and sleep efficiency (0.07), total sleep time (−0.01), or sleep period time (−0.08). The ESStr correlated with BMI (0.22) but not with age (0.08). ESStr correlated with the general productivity subscale (−0.22), activity level (−0.75), vigilance (−0.92), social outcome subscales (−0.62), and total FOSQtr (−0.72)	Difference in the ESStr score between group 1 (AHI < 15/hr) and group 2 (15 < AHI < 30/hr) and between group 1 and group 3 (AHI ≥ 30/hr): 10.62, 14, 16, respectively. Individuals with sleep-disordered breathing (12.6) > controls (3.6) for total ESStr. Those with sleep-disordered breathing had significantly higher ESStr scores than the control group		Turkish
Takegami et al., 2009 [76]: JESS	Development sample: 270 individuals (72 females; mean age of 42.1 years). Validation sample: 270 individuals (73 females; mean age of 40.6 years). Among the total sample, 143 patients (85 with OSAS, 54 with narcolepsy and 4 with idiopathic hypersomnia) with 43 females (mean age of 47.9 years)	A 1-factor solution (eigenvalue of 5.77) with factor loadings ranging from 0.47 to 0.76. JESS version with questions 9 (reading something while sitting in a chair) and 13 (while sitting and writing by hand) used instead of questions 1 and 8: 1-factor model with factor loadings ranging from 0.579 to 0.771 (in all participants), from 0.561 to 0.803 (in patients) and from 0.516 to 0.736 (in healthy people)	Cronbach’s alpha for the JESS total score was 0.85. The alpha coefficients were calculated for each 7-question scale made by eliminating one of the 8 questions: 0.82–0.84 range.Item-total correlation coefficients ranged from 0.53 to 0.69	1-week test-retest with ICC = 0.78		Daytime dysfunction domain score of the PSQI ranged from 0 to 3: JESS total score (6.8) for the group with 0 < JESS total score (13.6) for the group with 3. Patients (10.8) had a higher JESS score than did the healthy people (8.4). In 12 patients, a CPAP improvement was associated with a 3.67 point improvement in JESS score		Japanese
Zhang et al., 2011 [81]: mESS	A total of 122 patients with suspected SDB (119 cases of OSAS with AHI ≥ 5^−1^; 17 females, mean age of 46.7 years) and 117 normal healthy Chinese volunteers without SDB (27 females; mean age of 44.2 years)	mESS (substitution of item 8 of the original ESS with item 10 of 10-ISQ): in normal individuals 2-factor model (eigenvalues of 1.66 and 1.40, respectively) with F1 (items 1–4; factor loadings ranged from 0.55 to 0.74) and F2 (items 5–7 and item 10; factor loadings ranged from 0.33 to 0.71). In patients: 1-factor model (eigenvalue of 4.11) and factor loadings ranged from 0.64 to 0.79	Cronbach’s alpha of the original ESS was 0.83 and of mESS was 0.86 for patients. In normal volunteers Cronbach’s alpha for ESS was 0.40 and for mESS was 0.42. Item-total correlations for ESS ranged from 0.22 to 0.74 and for mESS ranged from 0.54 to 0.70 in patients. Item-total correlations for ESS ranged from 0.05 to 0.28 and for mESS ranged from 0.07 to 0.29 in normal individuals		Multiple regression test with mESS was, associated with AHI (β = 0.59) but not associated with age (β = 0.07), BMI (β = 0.02) and SpO_2_ < 90 (β = 0.15)	In 21 patients with OSAS before and after nasal CPAP sensitivity to change: 18.9 vs. 10.3. Difference between patients (11.0) and normal individuals (5.7), and difference between mild (5.7), moderate (8.8) and severe (13.8) OSA		Chinese (Mandarin)
Rosales-Mayor et al., 2012 [77]: ESS-VP and ESS-MPV	Comprehension phase: 60 Peruvians (normal subjects 37 females; mean age of 20.9 years). Test-retest phase: 75 Peruvians (normal subjects and 16 OSA patients; 37 females; mean age of 28.1 years). Psychometric properties phase: 207 Peruvians (normal subjects and 36 OSA patients; 100 females; mean age of 29.6 years)	ESS-PV: 2-factor model (eigenvalues of 3.322 and 1.030, respectively), explaining a total variance of 55%: F1 (items 1–3 and items 5–8 with factor loadings ranged from 0.587 to 0.735) and F2 (item 4 with factor loading of 0.608). ESS-MPV: 2-factor model (eigenvalues of 3.289 and 1.080, respectively), explaining a total variance of 55%: F1 (items 1, 2–8; factor loadings ranged from 0.564 to 0.748) and F2 (item 2 with factor loading of −0.623)	ESS-PV: Cronbach’s alpha was 0.790 and the item-total coefficient correlation ranged from 0.554 to 0.723. For people with and without OSA, the Cronbach’s alpha was 0.882 and 0.755, respectively. ESS-MPV in which item 8 was replaced by “sitting, while having lunch or dinner” (item A), “sitting writing on a paper or the computer” (item B), “standing and learning or not on a wall or furniture” (item C), “while in the bathroom sitting on the toilet” (item D): Cronbach’s alpha with item A was 0.774 and it improved when the item was removed (item-total correlation was 0.282). Cronbach’s alpha with item B was 0.786 and the item-total correlation was 0.390. Cronbach’s alpha with item C was 0.789 and the item-total correlation was 0.435. Cronbach’s alpha with item D was 0.771 and it improved when the item was removed (item-total correlation was 0.235)	ESS-PV: 23.6 day test-retest with correlations between first and second applications for each item ranged from 0.621 to 0.821 and with ICC = 0.841. The people with OSA with coefficient correlation was 0.846 and ICC = 0.845. For the non-OSA group the coefficient 0.833 and ICC = 0.833. 12.8 day test-retest with correlations between first and second applications for each item ranged from 0.680 to 0.901 and with ICC = 0.946. ESS-MPV: test-retest item A (correlation = 0.729; ICC = 0.726), scale + item A (correlation = 0.829; ICC = 0.828), item B (correlation = 0.485; ICC = 0.485), scale + item B (correlation = 0.806; ICC = 0.805), item C (correlation = 0.564; ICC = 0.563), scale + item C (correlation = 0.819; ICC = 0.819), item D (correlation = 0.698; ICC = 0.690), scale + item D (correlation = 0.826; ICC = 0.826). When the time between application < 14 days, the correlations ranged from 0.625 (item B) to 0.956 (scale + item A and scale + item D) and ICC ranged from 0.728 to 0.956	Coefficient correlations appeared between items 8 with each of the four alternative items: item 8 and A was 0.438, item 8 and B was 0.344, item 8 and C was 0.464, item 8 and D was 0.319.The coefficient between the modified scale with item A and ESS-PV was 0.988, between modified scale with item B and ESS-PV was 0.981, between the modified scale with item C and ESS-PV was 0.984, and between the modified scale with item D and ESS-PV was 0.983. The correlation between ESS-PV and ESS-MPV (item C) was 0.986 in the total population, 0.987 in the non-driving population and 0.985 in the driving population	ESS-PV: sensitivity to change after CPAP treatment (30.9 days) in 36 OSA patients from 13.44 in pre-treatment period to 5.55 in the treatment period (mean drop was 7.89). ESS-MPV: sensitivity to change after CPAP treatment in 9 OSA patients from 12.89 in the pre-treatment period to 5.55 in the treatment period (mean drop was 7.56)	-	Spanish (Peruvian)
Wu et al., 2012 [80]: ESS	A total of 3214 individuals (1678 females); mean age of 42 years	A 1-factor (eigenvalue of 2.78) with factor loading ranging from 0.44 to 0.69	Cronbach’s alpha was 0.80.Item-total correlation, with coefficients higher than 0.60 except in item 6 (0.39). Each item correlated with all other items: items 4 and 8 had the highest correlation (0.60)	Split-half reliability coefficient was 0.81	ESS scores were correlated with the score on the PF (−0.12), RP (−0.12), BP (−0.14), GH (−0.23), VT (−0.24), SF (−0.15), RE (−0.13), and MH (−0.23) subscales of the SF-36. Multiple linear regression analysis found that the scores of the PF (β = −0.13), RP (β = −0.12), BP (β = −0.13), GH (β = −0.24), VT (β = −0.25), SF (β = −0.16), RE (β = −0.13) and MH (β = −0.22) predicted ESS scores	No differences were found in ESS score among different regions, genders, ages, and occupation groups, while education (respondents with a medium level of education = 6.00) and BMI (18.5–23.9 = 6.00) did show differences, with lower ESS scores compared to those with education level and BMI above or below these levels. EDS prevalence (ESS > 10) was lower in respondents with a secondary or high school education, compared with those who had education levels of only primary school or below. EDS (ESS > 10) was more prevalent in respondents with BMI of 28.0 and above than those with normal BMI. The prevalence of EDS (ESS > 10) was higher among respondents with chronic diseases than in those not suffering from such diseases. EDS (ESS > 10) had lower scores in SF-36 subscales than non-EDS	ESS > 10	Chinese (Mandarin)
Baumgartel et al., 2013 [83]: ESS	A total of 337 pregnant women in their first trimester (PCA: mean age of 23.92 years; CFA: mean age of 24.7 years)	A 2-factor model explaining 50.59% of the total variance: F1 (items 1–5 and 7 with factor loadings from 0.492 to 0.747) and F2 (items 6–8 with factor loadings from 0.447 to 0.886)	Cronbach’s alpha was 0.751. Cronbach’s alpha of F1 was 0.743 and Cronbach’s alpha of F2 was 0.524. Excluding item 7, Cronbach’s alpha was 0.708, for F1 it was 0.706, and for F2 it was 0.488		The correlation between ESS-F1 and LOT was non-significant (0.094) as was that between ESS-F2 and LOT (0.075). Total ESS and LOT did not correlate (0.095). There was a correlation of 0.119 between F2 and item 5e (PSQI)			English (American)
Sadeghniiat Haghighi et al., 2013 [84]: ESS-IR	A total of 466 patients with suspected OSA identified by PSG (group 1 AHI < 5, 44 females, mean age of 39.89 years; group 2 5 ≤ AHI ≤ 15, 25 females, mean age of 45.00 years; group 3 15 ≤ AHI ≤ 30, 16 females, mean age of 47.93 years; group 4 AHI ≥ 30, 36 females, mean age of 48.74 years) and 41 patients with suspected narcolepsy with EDS identified by MSLT (11 females; mean age of 36.34 years)	In the PSG group, the 2-factor model (eigenvalues of 3.63 and 1.02) with 58.19% of variance explained: F1 (items 1–4 and items 7–8; factor loadings from 0.63 to 0.73) and F2 (items 5–6, factor loadings from 0.54 to 0.57). In the MSLT group, 2-factor model (eigenvalue of 4.53 and 1.18) with 61.50% of variance explained: F1 (items 1–3, 4–5 and 8; factor loadings from 0.71 to 0.84) and F2 (items 4, 7; factor loadings from 0.56 to 0.67)	Cronbach’s alpha in PSG group was 0.82 and in MSLT group was 0.88	2-to-4 weeks test-retest with ICC = 0.81	Correlations between item 6 and number of times patient fell asleep (0.358), item 3 and mean latency to sleep (−0.355), item 8 and mean latency to sleep (−0.382), item 4 and mean latency to REM (−0.474), item 6 and mean latency to REM (−0.475)	PSG group difference in total score (group1: 8.85, group2: 9.48, group3: 11.29, and group4:11.92). The same significant pattern was found for all ESS-IR items with the exclusion of item 5. Responsiveness of the ESS-IR in patients treated with CPAP: the ESS-IR score decrease before treatment (10.62) and 6–9 month after treatment (3.75)		Iranian
Sargento et al., 2015 [82]: ESS	A total of 176 healthy individuals (114 females; mean age of 38.68 years), 24 patients with mild to moderate OSA (4 females; mean age of 57.17 years) and 22 patients with severe OSA (7 females; mean age of 56 years)	A 2-factor model (eigenvalue: 3.194 and 1.418) explaining 57.65% of total variance: F1 (items 1–5 and 7; factor loadings from 0.32 to 0.78) and F2 (items 1, 3–4, 6 and 8; factor loadings from 0.53 to 0.80). Items 1, 3, 4 loaded on both factors and there was an interrelation between the two extracted factors (F1 and F2 = 0.700). A forced 1-factor solution was performed with commonalities ranging from 0.27 to 0.55, the total variance explained was 39.92%, and factor loadings ranged from 0.52 to 0.74. Rasch model: assumption of unidimensionality given that the analysis of residuals explained no more than 10% (9.2%) of the variance and the percentage of variance explained by the RM was over 20% (61%)	Cronbach’s alpha was 0.77. Item-total correlations ranged from 0.46 to 0.73 (items 6 and 8 were those with the lowest correlation with the total scale). Rasch model: item polarity showed a range from 0.43 to 0.72 in which all items were aligned in the same direction in the latent variable. Item separation reliability was 0.99, while person separation reliability was 0.78			Patients diagnosed with OSA (9.13) > healthy participants (7.4)		Portuguese (European)
Lapin et al., 2018 [85]: ESS	A total of 10,785 adult patients (5292 females); mean age of 49.6 years	A 1-factor solution (variance explained = 63.4%) with factor loadings from 0.673 to 0.844 (CFA confirmed). 2-factor model (variance explained = 68.4%): F1 (items 3, 4, 6, 8; factor loading from 0.475 to 0.903) and F2 (items 1, 2, 4, 5, 7; factor loadings from 0.351 to 0.866). 3-factor model (variance explained = 75.4%): F1 (items 3, 4, 6–8; factor loadings from 0.505 to 0.944), F2 (items 2–3; factor loadings from 0.676 to 0.879), F3 (item 5; factor loading = 0.995)	The ordinal alpha coefficient was 0.931 (ordinal alpha if item was deleted ranged from 0.916 to 0.930). Item-total correlation coefficients ranged from 0.590 to 0.822. Inter-item correlations were all significantly correlated with one another: item 1 × item 2 (0.74 or 0.73) and item 1 × item 3 (0.70 or 0.70); item 3 correlated with item 6 (0.75 or 0.76), item 7 (0.71 or 0.70) and item 8 (0.71 or 0.68). Item 5 correlated with item 6 (0.46 or 0.47) and item 8 (0.43 or 0.43)			Multigroup confirmatory factor analysis found measurement invariance across age groups, genders, and races, as well as for differing income levels, depression, and fatigue status. Sleepiness as measured by the ESS had a similar psychological meaning across all demographic subgroups		English (American)
Manzar et al., 2019 [79]: ESS	A total of 329 students (88 females): mean age of 20.96 years	A 1-factor model with incorporation of modification indices (co-varying error terms between item 7 and 8 with 0.20 and between item 4 and 5 with 0.22). Factor loadings ranged from 0.40 to 0.62	Cronbach’s alpha was 0.75 (Cronbach’s alpha if item was deleted ranged from 0.72–0.74. The item-total score ranged from 0.55 to 0.67. The inter-item correlation coefficient was between 0.12 and 0.40			Primary insomniacs (9.20) > normal sleepers (4.92). Those with primary insomnia obtained higher scores for each item than normal sleepers		Ethiopian

Abbreviations: SDS: sleep-disordered breathing; AHI: apnoea plus hypopnoea index; SaO_2_: saturation of oxygen; FOSQtr: Functional Outcomes of Sleep Questionnaire; BMI: body mass index; OSAS: Obstructive Sleep Apnea Syndrome; PSQI: Pittsburgh Sleep Quality Index; CPAP: Continuous Positive Airway Pressure; SpO_2_ < 90: percentage of sleep time with oxygen saturation below 90%; PF: physical functioning; RP: role limitation due to physical problems; RE: role limitation due to emotional problems; SF: social functioning; MH: mental health; VT: vitality; BP: bodily pain; GH: General Health perception; SF-36: Short Form Health Survey-36; PSG: polysomnography; MSLT: multiple sleep latency test; REM: rapid eyes movement; RM: Rasch model; LOT: Life Orientation Test; PSQI: Pittsburgh Sleep Quality Index.

## Data Availability

Data is contained within the article. In addition, the data and article presented in this study are available on request from the corresponding author.
